# Crosstalk of noradrenergic Ca^2+^ and cAMP signaling in astrocytes of the murine olfactory bulb

**DOI:** 10.1186/s12964-026-03055-z

**Published:** 2026-07-11

**Authors:** Jessica Sauer, Antonia Beiersdorfer, Franz Lennart Schmidt, Mathias Nordbeck, Oana Constantin, Daniela Hirnet, Christine E. Gee, Christian Lohr

**Affiliations:** 1 Division of Neurophysiology, University of Hamburg, Hamburg, Germany; 2 Center for Translational Neuromedicine, University of Copenhagen, Copenhagen, Denmark; 3 Sorbonne Université, Institut du Cerveau - Paris Brain Institute - ICM, Inserm, CNRS, APHP, Hôpital de la Pitié Salpêtrière, Paris, France; 4 Institute of Synaptic Neuroscience, ZMNH, University Medical Center Hamburg- Eppendorf, Hamburg, Germany

**Keywords:** Norepinephrine, Adenylyl cyclase, Phenylephrine, Cyclic adenosine monophosphate, Ca^2+^, astrocytes

## Abstract

Cyclic adenosine monophosphate (cAMP) and Ca²⁺ are ubiquitous second messengers that regulate gene expression, metabolism, and synaptic plasticity. Here, we identified a complex interplay between Ca²⁺ and cAMP signaling pathways in mouse olfactory bulb astrocytes. Norepinephrine (NE) elevated both Ca²⁺ and cAMP levels via α_₁_ and α_₂_ adrenergic receptors, whereas β receptors triggered only cAMP responses. The α_₁_ receptor agonist phenylephrine increased cAMP, but this effect was suppressed when Ca²⁺ elevations were blocked by Ca²⁺ depletion and removal of external Ca²⁺. We found that α_₁A_ and α_1D_ receptors are key targets for phenylephrine, acting through Ca²⁺/calmodulin-dependent adenylyl cyclases AC1 and AC3 downstream of α_₁_ receptor activation. Moreover, α_₂_ receptor stimulation raised Ca²⁺ levels, thereby stimulating cAMP production, yet also reduced forskolin-induced cAMP elevations, indicating that α_₂_ receptors can both inhibit adenylyl cyclase via G_i_ and stimulate AC1/AC3 via Ca²⁺ signaling. Together, these findings reveal intricate crosstalk between noradrenergic Ca²⁺ and cAMP signaling in olfactory bulb astrocytes mediated by all three adrenergic receptor subtypes.

**Supplementary Information:**

The online version contains supplementary material available at 10.1186/s12964-026-03055-z.

## Introduction

The locus coeruleus (LC) serves as the primary source of norepinephrine (NE) in the brain, where NE is synthesized in noradrenergic neurons by beta-hydroxylation of dopamine [1]. Notably, approximately 40% of all LC neurons project to the olfactory bulb (OB), a number that is tenfold greater than projections to any other region of the cerebral cortex [2]. NE and its effects on neural dynamics in the OB are essential for the formation of stable and specific olfactory memories [3]. NE enhances the contrast of weak neuronal sensory inputs in the OB by modulating mitral cell responses [4]. The NE system plays a pivotal role in both health and disease. In Parkinson´s Disease, for instance, not only does the substantia nigra degenerate, but so does the LC, with olfactory dysfunction being one of the early symptoms [5]. In addition, dysfunction of astrocytes is also implicated in the onset of neurological disorders [6]. Astrocytes are multifunctional glial cells that play a crucial role in maintaining homeostasis of the central nervous system (CNS), modulating information processing at molecular, cellular and whole network levels, and mediating both beneficial and detrimental effects during neuropathological diseases [7–9].

Noradrenergic receptors were initially classified into α and β receptors, the α receptors being further subdivided into α_1_ and α_2_ receptors [10, 11]. The α_2_ receptors are known to be coupled to G_i_ and inhibit adenylyl cyclase (AC) activity, whereas β receptors are coupled to G_s_, leading to AC stimulation, and α_1_ receptors are coupled to G_q_, stimulating PLC/IP_3_-dependent Ca^2+^ release from internal stores [12, 13]. Astrocytes throughout the brain exhibit Ca^2+^ transients following activation of α_1_ receptors [14]. However, astrocytic processes in the OB are located in close proximity to noradrenergic fibers and respond to NE application with Ca^2+^ signaling not only via α_1_ but also α_2_ receptors [15]. Besides Ca^2+^, cyclic adenosine monophosphate (cAMP) is another ubiquitous second messenger, synthesized from ATP by ACs. cAMP regulates a plethora of cellular functions such as gene transcription and translation. It plays significant roles in neurodevelopment and activity-dependent modifications of neuronal performance [16]. Astrocytes respond to several neurotransmitters and other signaling molecules with increases in cAMP, including NE [17, 18], dopamine [19], adenosine [20, 21] and lactate [22]. In astrocytes, cAMP signaling is involved in glucose metabolism [20, 22–24], local regulation of blood flow [25], exocytosis [26], synaptic plasticity [27] and behavior such as vigilance and memory formation [18, 28–30]. To date, ten isoforms of ACs have been characterized in mammals. Nine of them are membrane bound, activated by G_s_ and inhibited by G_i_ [31]. Three of these AC isoforms (AC1, AC3, and AC8) are Ca^2+^/CaM-stimulated, in addition to their activation by G_s_ [16, 32]. They could play a central role in the integration between Ca^2+^ and cAMP signaling, but direct evidence for this crosstalk is missing in astrocytes [33]. In addition, soluble adenylyl cyclase isoform 10 can be activated by Ca^2+^ and HCO_3_^−^, while being independent of G_s_ and G_i_ [34]. Ca^2+^-dependent adenylyl cyclases enable functional interaction between both second messenger systems, however, no detailed study of Ca^2+^/cAMP interactions on the single cell level exists in astrocytes.

In the present study, we investigated NE-induced cAMP and Ca^2+^ signals in astrocytes of the mouse OB, which is the first center of olfactory information processing [35]. Although NE-induced cAMP signaling has been extensively studied in various cortical regions and in the hippocampus, noradrenergic cAMP signaling remains underexplored in astrocytes of the OB, despite its significant innervation by projections from the LC [2, 18, 36, 37]. To investigate cAMP dynamics in the OB, we expressed the genetically encoded cAMP sensor Flamindo2 [38] in astrocytes and performed confocal cAMP imaging on acute olfactory bulb slices. Ca^2+^ was visualized by GCaMP6s and jRCaMP1a, respectively, expressed by astrocytes. Our results demonstrate that NE induces cAMP signals in OB astrocytes via α_1_, α_2_, and β receptors, while Ca^2+^ signals were triggered by α_1_ and α_2_ receptors only. Notably, rises in the cAMP concentration via activation of α receptors were dependent on Ca^2+^ release from internal stores such as the ER, whereas cAMP signals induced via β receptors were entirely Ca^2+^-independent. cAMP signals evoked by the α_1_ agonist phenylephrine (PE) were blocked by antagonists of α_1A_ and α_1D_ subtypes and were suppressed by inhibitors of AC1 and AC3. We conclude that NE directly induces an increase in cAMP by activation of β receptors coupled to G_s_, and indirectly by activation of α_1_ and α_2_ receptors leading to Ca^2+^ release from internal stores, thereby stimulating Ca^2+^/CaM-dependent AC1 and AC3.

## Results

### Norepinephrine evokes cAMP signals in astrocytes of the olfactory bulb

**Fig. 1:**
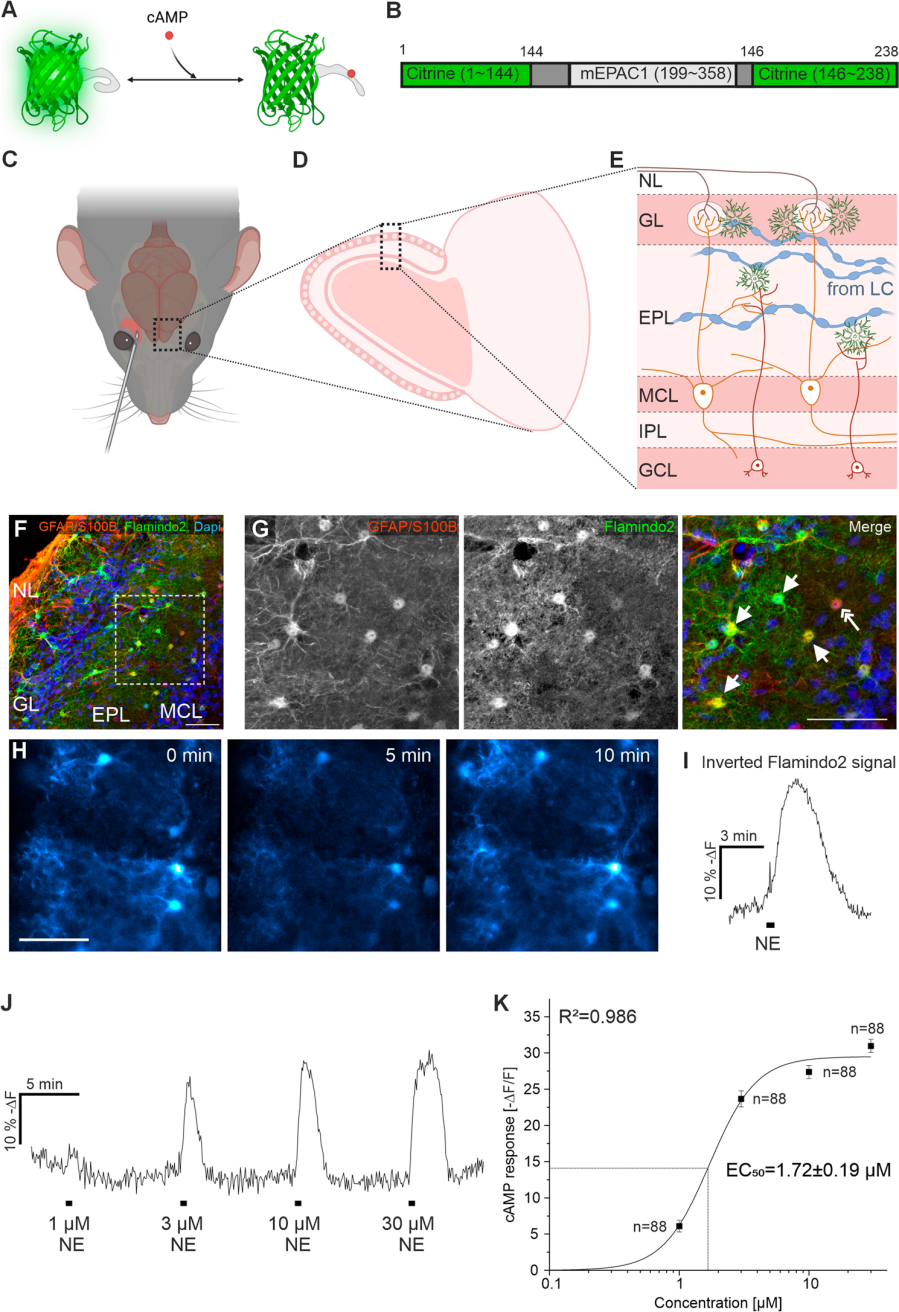
Norepinephrine induces cAMP signals in astrocytes of the olfactory bulb. **A** Schematic structure of Flamindo2. Note the decrease in fluorescence upon binding of cAMP. **B** Flamindo2 is derived from the yellow fluorescent protein (YFP) mutant, Citrine, and includes the cAMP binding site of mouse EPAC1 (exchange protein directly activated by cAMP) [38]. **C** Adeno-associated viral vectors (AAVs) carrying the Flamindo2 gene were injected into the retro-bulbar sinus. **D** Structure of a sagittal slice of the olfactory bulb. **E** Simplified cellular organization of the olfactory bulb: Axons of sensory neurons in the olfactory epithelium enter the nerve layer (NL) and form synapses with mitral cells in the glomerular layer (GL). Dendrites of mitral cells extend through the external plexiform layer (EPL). Cell bodies of mitral cells are located in the mitral cell layer (MCL), their axons proceeding through the internal plexiform layer (IPL) towards the olfactory cortex. The granular cell layer (GCL) contains the cell bodies of granular cells. Centrifugal fibers from the locus coeruleus (LC) containing varicosities innervate the olfactory bulb. Mitral cells are shown in orange color, granular cells in red, and astrocytes in green, centrifugal fibers in blue. **F** Immunostaining against Flamindo2 (anti-GFP; green) confirms the expression of Flamindo2 in olfactory bulb astrocytes labeled with anti-GFAP/anti-S100B (red). Nuclei were labeled with Dapi (blue). Scale bar: 50 μm **G** Magnified view of astrocytes in the olfactory bulb. Arrows indicating co-expression of Flamindo2 and GFAP/S100B, the double arrow indicates an astrocyte that is Flamindo2-negative. Scale bar: 50 μm. **H** Fluorescence of a Flamindo2-expressing olfactory bulb slice prior to (0 min) and during a 30 s application of 10 µM norepinephrine (NE) (5 min). Flamindo2 fluorescence recovered following NE washout (10 min). Scale bar: 50 μm. **I** Exemplary Flamindo2 fluorescence trace of a single astrocyte during NE application (10 µM, 30 s). Note the inverted vertical axis (-ΔF) applied to better reflect the changes in astrocytic cAMP concentration. **J** Astrocytic cAMP signals evoked by a range of NE concentrations. **K** Dose-response-curve of norepinephrine-induced cAMP signals. Data from 3 mice

To study NE-induced cAMP signaling in astrocytes of the OB we used the genetically encoded cAMP indicator Flamindo2 that consists of circularly permuted Citrine, a mutant of yellow fluorescent protein, fused with the cAMP binding domain of mouse EPAC1 (exchange protein directly activated by cAMP; Fig. 1A, B) [38]. We took advantage of AAV-driven Flamindo2 expression controlled by the astrocyte-specific gfaABC1D-promoter [39] after retro-bulbar injection and used brain slices including the olfactory bulb 3–6 weeks after virus injection (Fig. 1C-E). We confirmed that Flamindo2 is expressed exclusively in astrocytes by its co-localization with the astrocytic markers GFAP and S100B (Fig. 1F, G). Approximately 50% of the astrocytes in the glomerular and external plexiform layer of the OB were transduced. Conformational changes in Flamindo2 upon cAMP binding decreases fluorescence [38] (Fig. 1H). Note that subsequent Flamindo2 traces have been inverted (-ΔF) to represent increases in cAMP more intuitively (Fig. 1I; see [21]). Since no significant differences in NE-induced cAMP responses were found between the soma and cell processes, we analyzed only one region of interest in the following experiments, covering the soma and proximal processes (Fig. S1). The cAMP sensor Flamindo2 is pH-sensitive and NE-evoked fluorescence changes could be due to pH changes rather than cAMP changes [38]. Therefore, we tested the effect of NE application on intracellular pH in astrocytes loaded with the pH indicator pHrodo Red (Fig. S2A). Application of NE did not evoke changes in pH. In contrast, addition of 5 mM NH_4_Cl evoked an acidification that was accompanied by a decrease in Flamindo2 fluorescence, confirming that Flamindo2 fluorescence is pH-sensitive. Our results indicate that NE-induced Flamindo2 fluorescence signals were not mediated by pH changes but reflect cAMP changes (Fig. S2B). To elucidate the dose/response relationship of NE-induced cAMP signals in astrocytes, we applied different concentrations of NE (Fig. 1J). Application of 1 to 30 µM NE led to a dose-dependent increase in cAMP signals with an EC_50_ of 1.72 ± 0.19 µM (Fig. 1K; Table S1). All astrocytes expressing Flamindo2 responded to concentrations of 10 µM and higher, hence we applied 10 µM NE to induce maximal cAMP responses in OB astrocytes in further experiments.

### NE-induced cAMP signals are independent of neuronal activity and mediated via α_1_, α_2_, and β adrenergic receptors

**Fig. 2:**
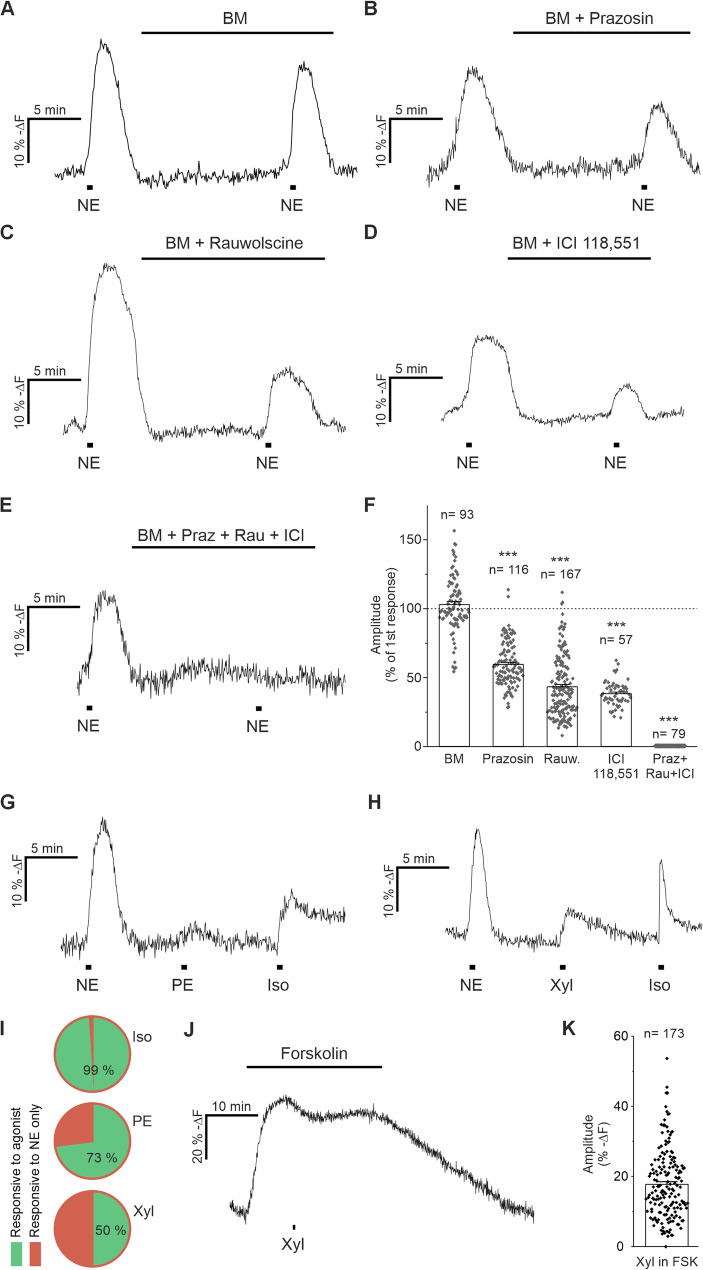
Norepinephrine-induced cAMP signals are independent of neuronal activity and mediated by α_1_, α_2_ and β adrenergic receptors. **A** Norepinephrine (NE)-induced cAMP signals in the presence of tetrodotoxin (TTX; first application) and TTX combined with glutamatergic and GABAergic inhibition in a blocker mix (BM) containing: 50 µM DAPV (NMDA receptors), 10 µM NBQX (AMPA receptors), 5 µM Gabazine (GABA_A_ receptors), 10 µM CGP 55,845 (GABA_B_ receptors). **B-E** Inhibition of NE-induced cAMP signals by the noradrenergic antagonists **B** prazosin (α_1_; 10 µM), **C** rauwolscine (α_2_; 0.5 µM), **D** ICI 118,551 (β; 15 µM), and **E** the combination of all three antagonists. **F** Quantification of amplitudes of cAMP transients normalized to the amplitude of the first NE application. ***; *p* < 0.001, Kruskal Wallis ANOVA and Dunn´s post hoc test. Data from 3 mice (prazosin), 3 mice (rauwolscine), 3 mice (ICI118,551) and 3 mice (Praz + Rau+ICI). **G** cAMP signals evoked by application of 10 µM NE, 100 µM phenylephrine (PE; α_1_ agonist), 100 µM isoprenaline (Iso; β agonist), and **H** 10 µM NE, 80 µM xylazine (Xyl; α_2_ agonist) and 100 µM Iso. **I** Fraction of astrocytes responding to Iso (total of 87 cells from 3 mice), PE (total of 30 cells from 3 mice) and Xyl (total of 66 cells from 3 mice). **J** Inhibitory effect of Xyl during elevation of cAMP levels upon adenylyl cyclase stimulation by 3 µM forskolin. **K** Amplitudes of xylazine-evoked decreases in cAMP during application of forskolin. Data from 4 mice

Since NE increases the excitability of neurons in the OB [4], we used 0.5 µM tetrodotoxin (TTX) to suppress action potentials in neurons contributing to NE-evoked cAMP signaling in astrocytes. NE was able to induce increases in cAMP when action potentials were blocked with TTX, however, the amplitude of NE-evoked cAMP signals decreased from 37.0 ± 1.3% -ΔF (*n* = 92) without TTX to 24.0 ± 0.8% -ΔF (*n* = 144) with TTX, indicating some contribution from neurons (Fig. S3A-C). To block any residual, TTX-insensitive neuronal effect, we assessed NE-induced cAMP responses in the additional presence of glutamatergic and GABAergic receptor inhibitors using a blocker mix (BM) consisting of TTX (0.5 µM), the NMDA receptor antagonist D-APV (50 µM), the AMPA receptor antagonist NBQX (10 µM), the GABA_A_ receptor antagonist gabazine (5 µM), and the GABA_B_ receptor antagonist CGP 55,845 (10 µM). During BM application, the mean amplitude reached 102 ± 3.8% of the response in TTX alone, which was not significantly different (*p* = 0.206; Wilcoxon), indicating that in the presence of TTX, NE directly increases astrocytic cAMP without a significant contribution from glutamate/GABA release from neurons or astrocytes that could affect astrocytic cAMP in an autocrine/paracrine manner (Fig. 2A).

We tested the involvement of α_1_, α_2_, and β adrenergic receptor subtypes by assessing the effect of subtype-specific antagonists on NE-induced cAMP signaling in astrocytes in the presence of TTX and glutamatergic/GABAergic antagonists. The antagonists prazosin (10 µM, α_1_ receptor) and rauwolscine (0.5 µM¸ α_2_ receptor) significantly decreased NE-evoked astrocytic cAMP responses to 60 ± 1.5% (*n* = 116; Fig. 2B) and 43 ± 1.7% (*n* = 167; Fig. 2C) of their respective controls. The β receptor antagonist ICI 118,551 (15 µM) reduced the NE-induced cAMP responses to 38 ± 1.2% of the control (*n* = 57; Fig. 2D), whereas a combination of all three antagonists completely blocked NE-evoked increases in cAMP (*n* = 79; Fig. 2E). These results, summarized in Fig. 2F, suggest that all three noradrenergic receptor subtypes contribute to astrocytic cAMP. Since glutamatergic/GABAergic antagonist did not result in additional inhibition of the NE-evoked cAMP response compared to TTX alone, all following experiments were performed with 0.5 µM TTX without glutamatergic/GABAergic antagonists. We next used subtype-specific agonists to verify the results obtained with the noradrenergic antagonists. 100 µM phenylephrine (PE; α_1_ receptor agonist), 80 µM xylazine (α_2_ receptor agonist) and 100 µM isoprenaline (β receptor agonist) increased cAMP in OB astrocytes (Fig. 2G, H). Isoprenaline induced the largest cAMP signals with an amplitude of 22.3 ± 0.7% -ΔF (*n* = 86 out of 87 cells), which were significantly larger than those induced by PE (5.6 ± 0.4% -ΔF, *n* = 22 out of 30 cells; *p* < 0.001) and xylazine (10.6 ± 0.7% -ΔF, *n* = 33 out of 66 cells; *p* < 0.001) (Fig. S4). Whereas 99% of the astrocytes responding to NE also increased cAMP in response to isoprenaline, only 73% and 48% of NE-responsive astrocytes responded to PE and xylazine, respectively (Fig. 2I). Since α_2_ receptors are known to couple to G_i_ and inhibit cAMP production, the cAMP increase mediated by the α_2_ receptor agonist xylazine was surprising. Interestingly, xylazine was also able to decrease 3 µM forskolin-induced cAMP production, indicating that α_2_ receptors in OB astrocytes couple to the G_i_ adenylyl cyclase-inhibiting pathway in addition to stimulating cAMP production (-17.7 ± 0.7% -ΔF from forskolin peak, *n* = 173, Fig. 2J, K).

### NE-induced cAMP signals partly depend on Ca^2+^ signals

**Fig. 3:**
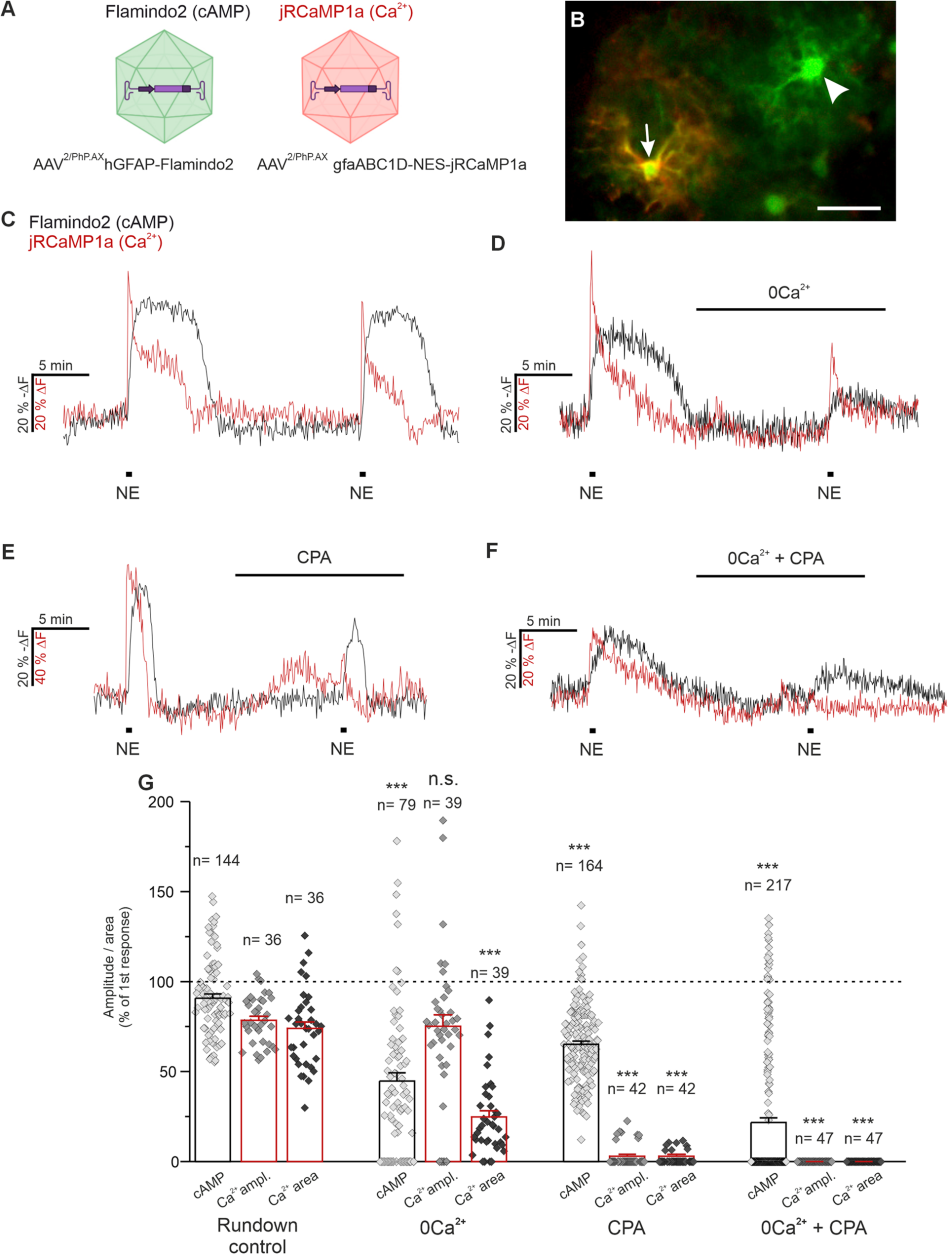
Norepinephrine-induced cAMP signals are partly Ca^2+^-dependent. **A** Astrocytes were co-transduced with AAVs carrying Flamindo2 or jRCaMP1a for simultaneous cAMP and Ca^2+^ measurements. **B** Astrocytes in an acute brain slice co-transduced with Flamindo2 and jRCaMP1a (arrow) and Flamindo2 alone (arrowhead). Scale bar: 30 μm. **C** cAMP (black) and Ca^2+^ signals (red) evoked by repetitive application of 10 µM norepinephrine (NE) to determine rundown effects. **D** Effect of calcium-free ACSF (0Ca^2+^), **E** Ca^2+^ store depletion by 20 µM cyclopiazonic acid (CPA), and **F** the combination of calcium-free ACSF and CPA on NE-evoked responses. **G** Quantification of cAMP and Ca^2+^ transients, compared to the corresponding response of the rundown experiment. For Ca^2+^ responses both the amplitude as well as the area under the curve were analyzed. Note that most data points for Ca^2+^ amplitude and Ca^2+^ area with CPA and 0Ca^2+^/CPA have a value of zero and lie on the baseline. ***p < 0.001, n.s.: not significant, Kruskal Wallis ANOVA and Dunn´s post hoc test. Some experiments were performed on mice only transduced with Flamindo2. Rundown control: 3 mice Flamindo2, 3 mice Flamindo2 + jRCaMP1a; 0Ca^2+^: 1 mouse Flamindo2, 3 mice Flamindo2 + jRCaMP1a; CPA: 3 mice Flamindo2, 3 mice Flamindo2 + jRCaMP1a; 0Ca^2+^ + CPA: 3 mice Flamindo2, 3 mice Flamindo2 + jRCaMP1a

Our results indicate that in OB astrocytes, not only β-adrenergic receptors but also α_1_ and α_2_ receptors trigger cAMP production. Previously, we showed that α_1_ and α_2_ receptors increase Ca^2+^ in OB astrocytes [15], raising the question whether NE-evoked Ca^2+^ activates ACs to increase cAMP. To investigate Ca^2+^ and cAMP signals simultaneously, we combined the red fluorescent Ca^2+^ indicator jRCaMP1a with Flamindo2 (Fig. 3A, B). Since repetitive application of NE leads to diminishing Ca^2+^ transients (rundown) [15], we first characterized the rundown of NE-induced cAMP signals (Fig. 3C). Initial applications of NE led to cAMP and Ca^2+^ signals with amplitudes of 24.0 ± 0.8% -ΔF for cAMP (*n* = 144) and 31.5 ± 2.3% ΔF for Ca^2+^ (*n* = 36; Fig. 3C). Second applications resulted in cAMP and Ca^2+^ transients that were 92 ± 1.9% of the first response for cAMP (*n* = 144; *p* < 0.001) and 78 ± 2.2% of the first response for Ca^2+^ (*n* = 36; *p* < 0.001). NE-evoked Ca^2+^ signals consisted of two components; a fast, transient Ca^2+^ rise reflecting Ca^2+^ release from intracellular stores, and a sustained plateau phase reflecting activation of store-operated Ca^2+^ entry upon store depletion, the latter being best described by the area under the curve [15]. Therefore, we additionally calculated the area under the curve of the Ca^2+^ signals, which was reduced to 74 ± 3.6% of the first response upon a second application of NE (*n* = 36; Fig. 3C, G). We used the second application of the rundown experiment in Fig. 3C as the control group and compared these Ca^2+^ and cAMP transients to the responses after pharmacological manipulation in the following experiments. To test whether Ca^2+^ is essential for cAMP signaling, we either omitted Ca^2+^ in the ACSF to prevent Ca^2+^ influx or inhibited the sarco-/endoplasmic reticulum Ca^2+^ ATPases (SERCA) with 20 µM cyclopiazonic acid (CPA) to deplete intracellular Ca^2+^ stores and suppress intracellular Ca^2+^ release. Ca^2+^ store depletion was visible by a moderate increase in cytosolic Ca^2+^ which was often accompanied by a slow increase in cAMP (Fig. S5A). After application of Ca^2+^-free ACSF for ten minutes, the peak Ca^2+^ response to NE was unaffected (75 ± 6.3% of the first response. *n* = 39; *p* > 0.05 vs. rundown control), while the area under the curve was significantly reduced to 25 ± 3.4% of the first response (*n* = 39; *p* < 0.001), confirming that the plateau phase but not the fast peak depends on Ca^2+^ influx (Fig. 3D, G).

Depleting extracellular Ca^2+^ significantly reduced cAMP signals to 45 ± 4.6% of the first response (*n* = 79; *p* < 0.001). Depleting intracellular Ca^2+^ stores by CPA almost entirely suppressed NE-evoked Ca^2+^ transients (*n* = 42) and significantly reduced cAMP signals to 65 ± 1.7% of the first response (*n* = 164; *p* < 0.001, Fig. 3E, G). When Ca^2+^-free ACSF and CPA were combined, NE-induced Ca^2+^ signals were completely eliminated (*n* = 47; Fig. 3F, G) whereas cAMP signals with an amplitude of 22 ± 2.6% of the first response persisted (*n* = 217; *p* < 0.001). Our results show that mechanisms whereby NE increases cAMP in OB astrocytes consist of a Ca^2+^-dependent and a Ca^2+^-independent component. β receptors are known to directly stimulate adenylyl cyclases by G_s_, suggesting that the Ca^2+^-independent pathway is stimulated by β receptors. Indeed, the β receptor agonist isoprenaline failed to induce Ca^2+^ signals (Fig. S5B, C). The cAMP responses to isoprenaline were not affected when Ca^2+^ stores were emptied by CPA, confirming that cAMP production by β receptors is Ca^2+^-independent (Fig. S5D-F).

### PE-induced cAMP signals are Ca^2+^-dependent

**Fig. 4:**
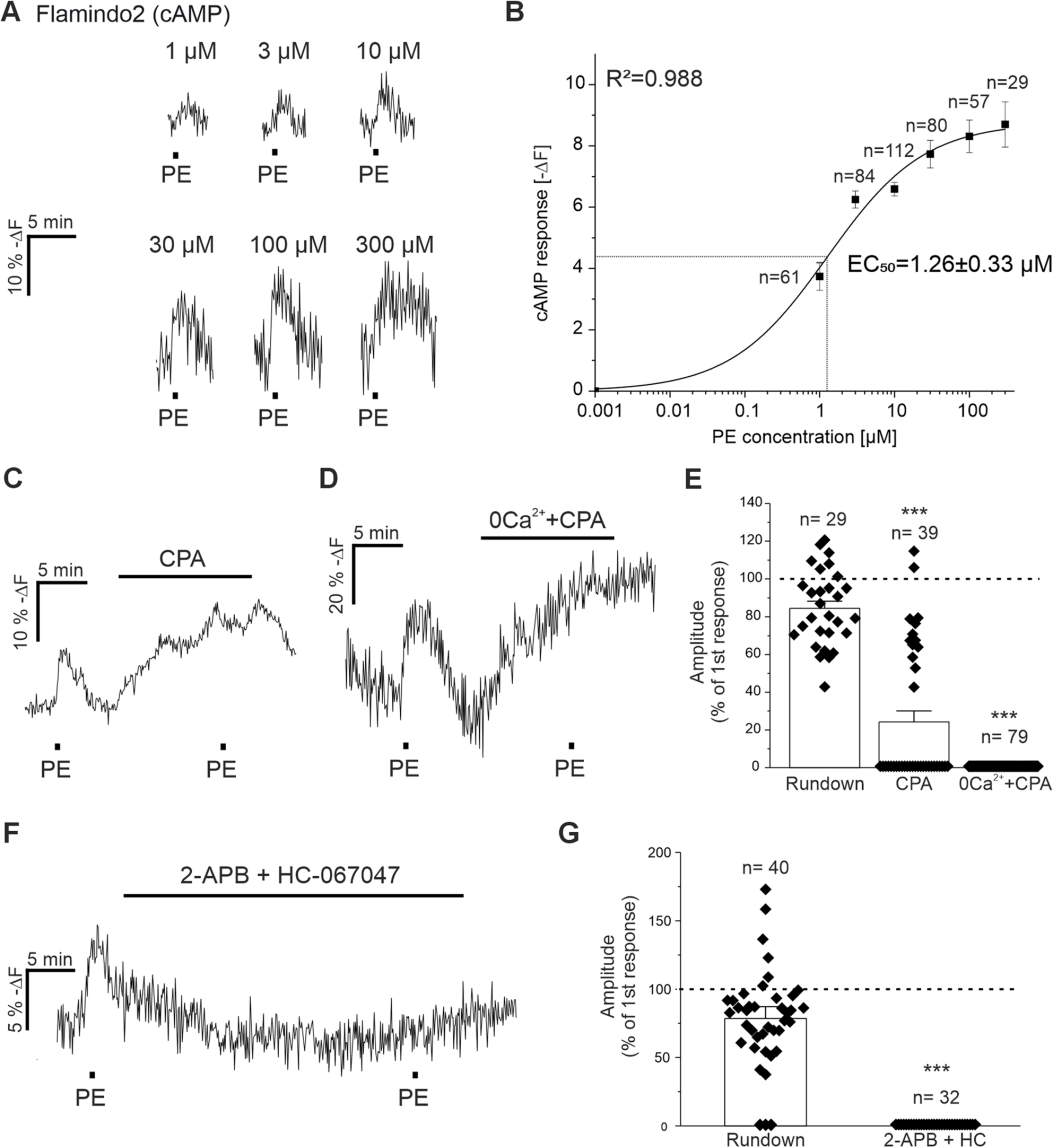
α_1_ receptor agonist phenylephrine induces Ca^2+^-dependent cAMP signals. **A** cAMP signals in olfactory bulb astrocytes evoked by phenylephrine (PE) at concentrations ranging from 1 µM to 300 µM. **B** Dose-response curve of PE-induced cAMP signals. Experiments from 6 mice. **C** PE-induced cAMP signals in the presence of 20 µM cyclopiazonic acid (CPA) and **D** the combination of calcium-free ACSF (0Ca^2+^) and CPA. **E** CPA and CPA/0Ca^2+^ significantly reduced the amplitude of PE-evoked cAMP signals when compared to the corresponding rundown experiment (***; *p* < 0.001, Kruskal Wallis ANOVA and Dunn´s post hoc test). 3 mice for rundown experiments, 3 mice for CPA, 4 mice for 0Ca^2+^+CPA. **F** PE-induced cAMP signals were suppressed in the presence of the IP_3_ receptor inhibitor 2-APB (100 µM) and the TRPV4 inhibitor HC-067047 (1 µM). **G** Quantification of the effect of 2-APB + HC-067047. ***; *p* < 0.001, Mann-Whitney U-Test; 7 mice for rundown long, 4 mice for 2-APB + HC

Activation of α_1_ and α_2_ receptors increases Ca^2+^ in OB astrocytes [15] and canonically they are not linked to G_s_ proteins, suggesting that the Ca^2+^-dependent increases in cAMP in our study is mediated by α_1_ and α_2_ receptors. Ca^2+^-dependent cAMP signaling in OB astrocytes is best studied by selective activation of α_1_ receptors by, e.g., PE, since α_2_ receptors both increase cAMP and decrease adenylyl cyclase activity in OB astrocytes (Fig. 2H-K). PE at concentrations between 1 and 300 µM led to dose-dependent cAMP signals with amplitudes ranging from 3.7 ± 0.5% -ΔF (1 µM PE) to 8.7 ± 0.5% -ΔF (300 µM PE; *n* = 29–112; Fig. 4A; Table S2). We calculated the EC_50_ to be 1.26 ± 0.33 µM (Fig. 4B). For further experiments, we used 10 µM PE to elicit robust sub-saturating cAMP signals. We applied PE twice in rundown experiments with intervals of 10 min (RD short) and 30 min (RD long) between the cAMP signals and compared these rundowns to any further experiment (Fig. S6A-D). A second application of PE evoked cAMP responses in RD short of 84 ± 3.7% of the first application (*n* = 29) and in RD long of 79 ± 5.7% of the first application (*n* = 40).

To investigate the Ca^2+^ dependency of PE-induced cAMP signals, we blocked SERCA pumps with 20 µM CPA to deplete Ca^2+^ stores (Fig. 4C). After depletion of Ca^2+^ stores by incubation of CPA for 10 min, PE-induced cAMP signals were significantly smaller compared to the corresponding rundown (RD short; *p* < 0.001) and averaged 24 ± 5.8% (*n* = 39) of the first response, while PE-induced cAMP signals were entirely suppressed in the presence of CPA and Ca^2+^-free ACSF (0Ca^2+^/CPA; Fig. 4C-E). Forskolin-induced cAMP signals, in contrast, were not affected by CPA, showing that CPA does not directly inhibit cAMP production by, e.g., blocking adenylyl cyclases (Fig. S7A-C). To confirm the involvement of intracellular Ca^2+^ release, we inhibited IP_3_ receptors with 100 µM 2-APB, which blocks both IP_3_ receptors and store-operated Ca^2+^ entry [40, 41]. As 2-APB at concentrations above 50 µM also stimulates TRPV4 channels and Ca^2+^ oscillations in OB astrocytes [42], we co-applied 1 µM of the selective TRPV4 antagonist HC-067047 with 2-APB for 30 min [15]. The PE-induced increase in cAMP vanished completely after blocking IP_3_ receptors, indicating that Ca^2+^ release mediated by IP_3_ receptors is required for cAMP production evoked by α_1_ receptors (*n* = 32; Fig. 4F, G). In conclusion, our results demonstrate that PE-induced Ca^2+^ signals depend on the PLC/IP_3_ pathway as well as Ca^2+^ release from the endoplasmic reticulum and trigger increases in cAMP.

### The α_1A_ and α_1D_ receptor subtypes increase Ca^2+^ and cAMP

**Fig. 5:**
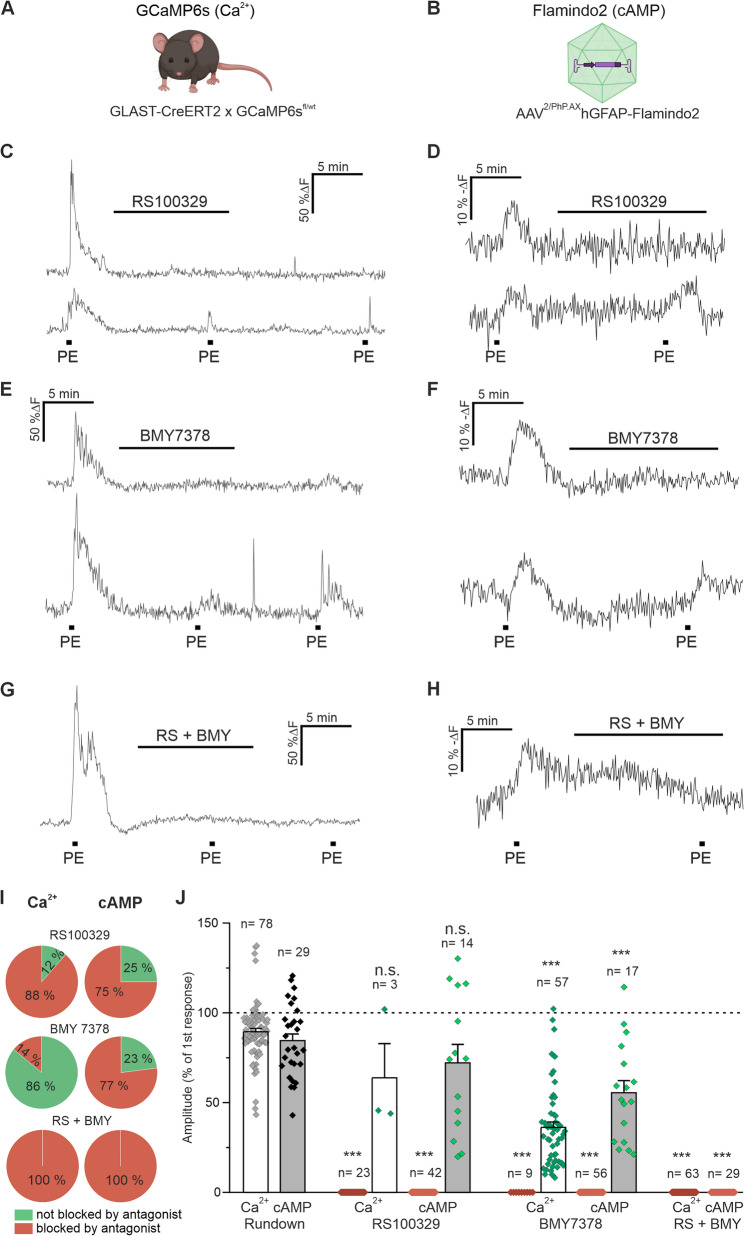
Phenylephrine induces Ca^2+^ and cAMP signals via α_1A_ and α_1D_ receptor subtypes. **A** To visualize Ca^2+^ signaling in olfactory bulb astrocytes, GCaMP6s was expressed under control of the promoter of the astrocyte-specific glutamate-aspartate transporter (Glast). **B** Flamindo2 was introduced into wild type mice by AAV to visualize cAMP signaling. **C** Effect of RS100329 (α_1A_; 1 µM) on phenylephrine (PE)-induced Ca^2+^ signals and **D** cAMP signals. RS100329 entirely inhibited Ca^2+^ and cAMP signals in a subpopulation of astrocytes (upper traces), while Ca^2+^ and cAMP signals remained in other astrocytes (lower traces). **E** BMY7378 (α_1D_; 1 µM) blocked Ca^2+^ signals and **F** cAMP signals in some astrocytes (upper traces) and reduced Ca^2+^ and cAMP signals in the remaining astrocytes (lower traces). **G** The combination of RS100329 and BMY entirely inhibited PE-induced Ca^2+^ and **H** cAMP signals. **I** Fraction of responsive vs. non-responsive astrocytes in the presence of RS100329, BMY7378 and the combination of both. **J** Analysis of PE-induced Ca^2+^ and cAMP signals. Note that in the presence of RS100329 as well as BMY7378 two subpopulations of astrocytes could be identified, one with Ca^2+^ and cAMP transients entirely blocked by the antagonists and one with residual Ca^2+^ and cAMP transients in the presence of the antagonists. ***p < 0.001, n.s.= not significant; Kruskal Wallis ANOVA and Dunn´s post hoc test. Ca^2+^ imaging: 4 mice for rundown, 3 mice for RS100329, 3 mice for BMY7378, 3 mice for RS100329 + BMY7378. cAMP imaging: 3 mice for rundown, 4 mice for RS100329, 3 mice for BMY7378, 4 mice for RS100329 + BMY7378

The α_1_ receptors are classified into the subtypes α_1A_, α_1B_, and α_1D_ [43, 44]. For further investigation of Ca^2+^ and cAMP signals induced by PE-application, we used specific antagonists for the different α_1_ receptor subtypes. Due to the relatively low co-expression rate of combined jRCaMP1.0 and Flamindo2 AAVs in the previous experiments, we performed the following experiments for Ca^2+^ and cAMP imaging separately, using GLAST-CreERT2 x GCaMP6s^flox^ mice for Ca^2+^ and AAV carrying Flamindo2 for cAMP (Fig. 5A, B). Application of PE evoked Ca^2+^ transients in 95% of all astrocytes (*n* = 73 out of 77), i.e. astrocytes that responded to application of NE, indicating that the majority of OB astrocytes express α_1_ receptors linked to Ca^2+^ signaling (Fig. S8A). PE-evoked cAMP signals, in contrast, only occurred in 53% of astrocytes (*n* = 71 out of 133) in this set of experiments, suggesting that α_1_ receptors are not linked to cAMP production in some cells (Fig. S8B, C). To study the contribution of α_1A_ receptors, we used the α_1A_-specific antagonist RS100329, which had a bimodal effect on both Ca^2+^ and cAMP signals. 1 µM RS100329 entirely blocked PE-induced Ca^2+^ signals in 88% of astrocytes (*n* = 23 out of 26), while Ca^2+^ signals were only weakly reduced in the remaining 12% of the astrocytes and, on average, did not differ significantly from the rundown experiment (*p* > 0.05; *n* = 3 out of 26; Fig. 5C, I and Fig. S9A, B). In addition, RS100329 entirely blocked PE-induced cAMP signals in 75% of the astrocytes (*n* = 42 out of 56) and resulted in PE-induced cAMP signals that did not differ significantly from the rundown experiment in the remaining 25% of astrocytes (*n* = 14 out of 56; Fig. 5D, I). The α_1D_ receptor antagonist BMY7378 also had a bimodal effect. 1 µM BMY7378 completely blocked PE-induced Ca^2+^ signals in 14% of the cells (*n* = 9 out of 66) and reduced Ca^2+^ signals to 36 ± 3.0% of the initial amplitude in 86% of the cells (*p* < 0.001; *n* = 57 out of 66; Fig. 5E, I). BMY7378 blocked PE-induced cAMP signals in 70% of the cells (*n* = 56 out of 73) and reduced cAMP signals to 55 ± 6.9% of the initial amplitude in the remaining 30% of the cells (*p* = 0.171; *n* = 17 out of 73; Fig. 5F, I). Combination of both antagonists completely abolished both PE-induced Ca^2+^ (*n* = 63; Fig. 5G, I) and cAMP signals (*n* = 29; Fig. 5H, I). The complete inhibition of the second messengers by combination of α_1A_ and α_1D_ antagonists indicates that α_1B_ does not play a significant role in OB astrocytes. In summary, our results show that α_1_ receptors, including α_1A_ and α_1D_, induce Ca^2+^ signaling in virtually all astrocytes of the OB, while cAMP signals are evoked in a subpopulation of astrocytes (Fig. 5J).

### Ca^2+^-induced cAMP signals are mediated by AC1 and AC3

**Fig. 6:**
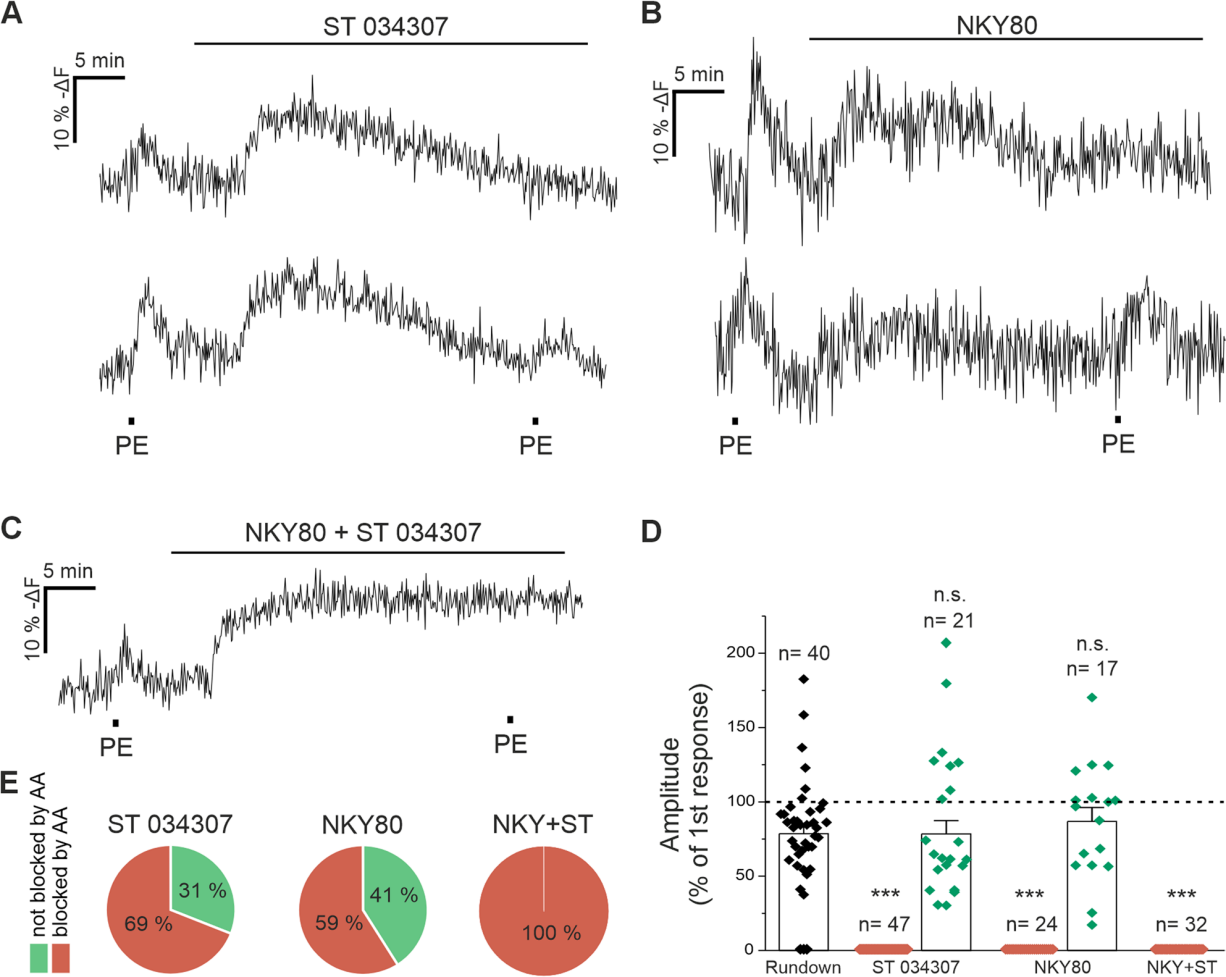
Phenylephrine-induced cAMP signals are mediated via adenylyl cyclase subtypes 1 and 3. **A** Effect of the AC1 blocker ST 034307 (10 µM) on phenylephrine (PE)-induced cAMP signals. ST 034307 entirely blocked cAMP transients in a subpopulation of astrocytes (upper trace), but had no significant effect on the cAMP transients in the remaining astrocytes (lower trace). **B** Effect of the AC3-blocker NKY80 (500 µM) on PE-induced cAMP signals. In some astrocytes, NKY80 entirely inhibited cAMP transients (upper trace), while in the remaining astrocytes it had no significant effect on cAMP transients (lower trace). **C** Combination of ST 034307 and NKY80 completely blocked PE-induced cAMP signals. **D**. Fraction of astrocytes responding to NE vs. not responding to NE in the presence of ST 034307, NKY80 and the combination of ST 034307 and NKY80. **E** Analysis of the effects of the adenylyl cyclase inhibitors on PE-evoked cAMP signals. ***p < 0.001, n.s.= not significant; Kruskal Wallis ANOVA and Dunn´s post hoc test. 7 mice for rundown, 5 mice for ST 034307, 5 mice for NKY80, 3 mice for ST 034307 + NKY80

Our results show that an increase in Ca^2+^ can lead to an increase in cAMP in OB astrocytes, suggesting the involvement of Ca^2+^-activated adenylyl cyclases. Three out of the nine transmembrane AC isoforms can be activated by Ca^2+^/CaM: AC1, AC3 and AC8 and all are expressed in OB cells, including astrocytes [31]. To test the contribution of Ca^2+^-activated AC1 and AC3 isoforms to cAMP signaling in OB astrocytes, we used the AC1-specific antagonist ST 034307, as well as NKY80, an antagonist of several adenylyl cyclases including AC3, while there is no specific antagonist for AC8 available [45, 46]. Blockage of AC1 with 10 µM ST 034307 completely inhibited PE-induced cAMP responses in 69% of the astrocytes (*n* = 47 out of 68), while in the remaining 31% of the astrocytes, the response did not differ significantly from the rundown experiment (*n* = 21 out 68; *p* = 1; Fig. 6A, D). In addition, NKY80 (500 µM) completely blocked cAMP signals in 59% of the cells (*n* = 24 out of 41), whereas in the remaining 41% of the cells, the cAMP signal was not statistically different from the rundown experiment (*n* = 17 out of 41; *p* = 1; Fig. 6B, D). The combined blockage of AC1 and AC3 led to a total inhibition of the PE-induced cAMP signal (*n* = 32; Fig. 6C-E). In our experiments, application of the AC inhibitors itself led to an increase in the cAMP concentration, which might result from them being a positive modulator of Ca^2+^-independent AC2 [46]. In conclusion, our results suggest that stimulation of α_1_ receptors by PE induces Ca^2+^-dependent cAMP signals in OB astrocytes via activation of AC1 and AC3.

## Discussion

In this study, we investigated the mechanisms by which NE evokes cAMP signaling in astrocytes of the glomerular and external plexiform layers of the OB. Our data demonstrate that NE induces cAMP generation via all three adrenergic receptor classes, i.e., α₁, α₂, and β receptors. While β receptors evoke cAMP signals in all OB astrocytes in a Ca^2+^-independent manner by direct stimulation of G_s_-proteins, α₁-mediated cAMP responses as well as α₂-mediated cAMP responses occur in a subpopulation of astrocytes and require intracellular Ca²⁺ rises to activate Ca²⁺-sensitive adenylate cyclases AC1 and AC3. Since these ACs are stimulated by both G_s_ and Ca^2+^, α and β receptor activation is required to fully drive cAMP production. These findings highlight a complex, receptor- and Ca²⁺-dependent regulation of cAMP signaling that has not been described in astrocytes before.

### NE induces cAMP signals in astrocytes of the olfactory bulb via α_1_, α_2_, and β receptors

We determined an EC₅₀ of approximately 2 µM for NE-induced cAMP responses in OB astrocytes and used 10 µM for all further experiments, a concentration also applied in previous studies addressing Ca^2+^ signaling in OB astrocytes and excitability of OB neurons [15, 47]. This concentration is one to two orders of magnitude higher than published EC_50_ values, however, in brain slices the effective concentration of NE deep in the tissue is much lower than in the experimental solution due to diffusion barriers and uptake of NE by cells [48]. Importantly, suppression of neuronal activity and synaptic transmission only partly reduced NE-induced cAMP signals, suggesting that the remaining effect is a direct consequence of NE acting on astrocytes, independent of neuronal input. Astrocytes are known to participate in the “glutamate amplifies noradrenergic effects” (GANE) mechanism by releasing glutamate that triggers neuronal activity and hence further NE release [14, 49]. As inhibiting neuronal activity decreased NE-evoked rises in cAMP, such a feedback loop also appears to exist in the olfactory bulb. We found that agonists for α₁, α₂, and β receptors each induced cAMP elevations, indicating that all three receptor types contribute to cAMP signaling under resting conditions, which is in contrast to, e.g., hippocampal astrocytes in which β receptors increase cAMP, and only α₁ receptors increase Ca^2+^ [18]. Given NE’s binding affinities at these receptors (α₂ > α₁ > β; [50]), even low NE levels could recruit α₂ and α₁ receptors, while higher concentrations are needed to activate β receptors.

An unexpected result was the increase in cAMP evoked by α₂ receptor activation. Astrocytes in the cortex show opposing regulation of cAMP by α₂ and β receptors, starting from astrocytic cAMP resting state [51], while in our experiments the α₂ agonist xylazine decreased cAMP only when ACs were pre-activated with forskolin. Hence, α₂ receptors bimodally regulate cAMP signals depending on the activation state of the astrocyte, increasing cAMP at resting cAMP levels and decreasing cAMP when ACs are already active and cAMP levels are elevated.

### NE-induced and PE-induced cAMP signals differ in their Ca^2+^ dependency

Previous work has shown NE-induced Ca²⁺ transients in astrocytes via α₁ and α₂ receptors, but not β receptors [15], and we now extend these findings by demonstrating that NE-induced Ca^2+^ signals increase cAMP. In the absence of beta activation and hence G_s_-dependent AC stimulation (ICI118,551, Fig. 2F), only Ca^2+^-dependent AC stimulation remains that accounts for 40% of the NE-evoked cAMP signal, suggesting a contribution of Ca^2+^-independent cAMP production of 60%. However, when suppressing Ca^2+^-dependent cAMP production to isolate Ca^2+^-independent cAMP production (0Ca^2+^/CPA, Fig. 3G), only 20% of the response was found to be Ca^2+^-independent. This discrepancy leads to the conclusion that both G_s_ and Ca^2+^ act synergistically to fully activate AC1 and AC3 in OB astrocytes. We cannot rule out that β receptors additionally stimulate other ACs that are not Ca^2+^-dependent. The small NE-evoked cAMP response in the nominal absence of Ca^2+^ signaling, however, suggests a minor contribution of such Ca^2+^-independent ACs. In contrast to NE-evoked cAMP signals, PE-induced α₁-mediated cAMP signals were entirely dependent on Ca²⁺ signaling. Although CPA alone did not fully block these responses, the combination of CPA with Ca²⁺-free ACSF as well as IP_3_ receptor antagonism entirely abolished cAMP generation. These results suggest that the initial ER-derived Ca²⁺ release and subsequent store-operated Ca²⁺ entry (SOCE) are essential for PE-induced AC activation. Interestingly, while activation of α₁ receptors have previously been associated primarily with Ca²⁺ signaling in astrocytes of various brain regions, as shown in vivo in the cerebral cortex [18], cAMP responses to α₁ receptor activation have been reported in peripheral systems such as the uterine smooth muscle cells [52]. Our data show that OB astrocytes also have α₁ receptor coupling to increases in cAMP via Ca^2+^-dependent AC1 and AC3. This highlights functional diversity in α₁-dependent signaling across brain regions and the linkage of the two second messenger pathways in astrocytes.

### PE-induced cAMP signals require α₁_A_ and α₁_D_ receptors and subsequent activation of AC1 and AC3

We identified both α₁_A_ and α₁_D_ noradrenergic receptors as key mediators of PE-induced Ca²⁺ and cAMP signals in OB astrocytes. However, there is a discrepancy between Ca^2+^ and cAMP signaling regarding the cell fractions that are sensitive to inhibition of α₁_A_ versus α₁_D_ receptors (Fig. 5I). Blocking α₁_A_ receptors completely inhibited PE-induced Ca²⁺ signals in 88% of cells, whereas blocking α₁_D_ receptors entirely blocked PE-induced Ca²⁺ signals in only 14% of the cells, suggesting that both astrocyte populations complete each other, with α₁_A_ being sufficient to elicit a Ca^2+^ rise in the majority of astrocytes. PE-induced cAMP signals were entirely blocked by inhibition of α₁_A_ receptors in 75% of the cells, a fraction similar to that found in Ca^2+^ imaging experiments. Inhibition of α₁_D_ receptors by BMY 7378 suppressed cAMP signals in about 80% of the astrocytes, a cell fraction much larger compared to the fraction of astrocytes in which Ca^2+^ signaling was entirely inhibited by BMY 7378. However, Ca^2+^ signals in astrocytes that were not entirely blocked by BMY 7378 were strongly attenuated, indicating that α₁_D_ receptors, while not essential to evoke Ca^2+^ signals, augment Ca^2+^ signals induced by α₁_A_ receptors. Consequently, attenuated PE-evoked Ca^2+^ signals in the presence of BMY 7378 were insufficient to stimulate Ca^2+^-dependent ACs and failed to elicit measurable cAMP signals in the majority of astrocytes. Our data identified AC1 and AC3, two ACs that are activated by both G_s_ and Ca^2+^, as the primary ACs responsible for PE-induced cAMP production using the selective AC1 inhibitor ST034307 and the non-specific AC inhibitor NKY80. NKY80 is known to mainly inhibit AC5 and additionally AC2, AC3 and AC6, however, AC2, AC5 and AC6 are not activated via Ca^2+^ and thus not involved in Ca^2+^-dependent cAMP signals as described in the present study [45, 46, 53]. Soluble adenylyl cyclase AC10 is highly expressed in astrocytes in some brain regions such as the hippocampus and is Ca^2+^-activated [23, 54], hence it could mediate PE-induced cAMP rises. However, neither ST034307 nor NKY80 have been reported to inhibit AC10, and the complete inhibition of PE-evoked cAMP signals by combined ST034307 and NKY80 strongly suggests that not AC10, but AC1 and AC3 are the main Ca^2+^-activated adenylyl cyclases in OB astrocytes, and also argues against a major contribution of AC8. Since no AC8-specific antagonist exists, however, direct evidence for or against a contribution of AC8 in Ca^2+^-dependent cAMP production is lacking in our study. Indeed, prior studies emphasized AC8 expression in the entire OB by in situ hybridization and demonstrated expression of AC1 and AC8 by single-cell RNAseq in OB astrocytes [31, 55, 56]. While AC8 appears to be expressed, our data suggests that AC1 and AC3 are more relevant in OB astrocytes and AC8 might play a more prominent role in other cell types.

### Conclusion and implications

Together, our findings provide a comprehensive view of NE-induced cAMP signaling in OB astrocytes and reveal receptor-specific and Ca²⁺-dependent as well as Ca^2+^-independent signaling pathways that act synergistically to fully activate cAMP production. NE activates astrocytic cAMP production via α₁, α₂, and β receptors, with distinct downstream mechanisms. Increases in cAMP evoked by β receptors were Ca^2+^-independent and employ the canonical G_S_ pathway, whereas PE-induced responses rely exclusively on intracellular Ca²⁺ signaling and require combined α₁_A/D_ receptor activation for full activation of Ca²⁺-dependent AC1/AC3. Activation of α₂ receptors also elicits both Ca^2+^ and cAMP rises, suggesting they employ the same signaling mechanism involving Ca^2+^-dependent stimulation of AC1 and AC3, however, when cAMP levels are elevated, α₂ receptors inhibit cAMP production by G_i_. These results underscore the complex integration and tuning of cAMP and Ca²⁺ signaling in astrocytes and, in consideration of the importance of both Ca^2+^ and cAMP for metabolic interaction as well as synaptic plasticity, indicate that both second messenger systems strongly interact to optimize neuronal performance.

## Materials and methods

### Animal handling

Mice were held in the animal facility of the Institute of Cell and Systems Biology (University of Hamburg, Hamburg, Germany) at a 12 h light/12 h dark cycle with food and water *ad libitum.* Animal handling and all experiments were approved by the local authorities (Behörde für Justiz und Verbraucherschutz, Lebensmittel und Veterinärwesen Hamburg; N010/2022) and followed German and European laws. To express the cAMP sensor Flamindo2 in astrocytes, C57Bl/6J wild type mice of both sexes at an age range from 4 to 16 weeks at the time of injection were used. They were injected with endotoxin-free recombinant adeno-associated viruses AAV^2/PhP.eB^hGFAP-Flamindo2 or AAV^2/PhP.AX^hGFAP-Flamindo2 [21, 38]. The capsid plasmid PhP-AX (Addgene #195218) was kindly provided by Vivianna Gradinaru and Xinhong Chen [57] and the capsid plasmid PhP.eB (Addgene #103005) was a gift from V. Gradinaru [58]. All AAVs were produced at the vector facility of the University Medical Center Hamburg-Eppendorf (Hamburg, Germany). 70 µl virus suspension containing 1*10^11^ vg was injected i.v. into the retro-bulbar sinus under isoflurane-anesthesia (Fig. 1A). Experiments were performed 3 to 6 weeks after injection. For experiments with simultaneous cAMP and Ca^2+^ imaging, 1*10^11^ vg AAV^2/PhP.AX^ gfaABC1D-NES-jRCaMP1a (addgene # 171120, Lohr et al., 2021) was co-injected to image astrocytic Ca^2+^. For Ca^2+^ imaging without cAMP imaging, GLAST-CreERT2 x GCaMP6s^fl/wt^ mice [59, 60] at an age range between 6 and 21 weeks were injected intraperitoneal three days in a row with tamoxifen (Carbolution Chemicals, St. Ingbert, Germany), 10 mg/ml in Miglyol (Caelo, Hilden, Germany) at 10 µl/g mouse weight. Imaging was conducted between 7 and 12 days after the first tamoxifen injection.

### Solutions and drugs

Artificial cerebrospinal fluid (ACSF) used during experiments contained (mM): 120 NaCl, 26 NaHCO_3_, 1 NaH_2_PO_4_, 2.5 KCl, 2.8 D-glucose, 2 CaCl_2_, 1 MgCl_2_. For the preparation of the OBs and brain slicing, Ca^2+^-reduced ACSF was used containing (in mM): 83 NaCl, 26.2 NaHCO_3_, 1 NaH_2_PO_4_, 2.5 KCl, 70 sucrose, 20 D-glucose, 0.5 CaCl_2_, 2.5 MgSO_4_. For experiments in Ca^2+^-free conditions (0Ca^2+^), ACSF was used containing (mM): 120 NaCl, 26 NaHCO_3_, 1 NaH_2_PO_4_, 2.5 KCl, 2.8 D-glucose, 3 MgCl_2_, 0.5 EGTA. All components were obtained from Carl Roth (Karlsruhe, Germany).

The following drugs were used: (R)-(-)-Phenylephrine hydrochloride (3-[(1R)-1-hydroxy-2-(methylamino)ethyl]phenol; hydrochloride; Abcam, Cambridge, United Kingdom), 2-APB (2-diphenylboranyloxyethanamine; Calbiochem, Darmstadt, Germany), BMY7378 (8-[2-[4-(2-methoxyphenyl)piperazin-1-yl]ethyl]-8-azaspiro[4.5]decane-7,9-dione; dihydrochloride; Hello Bio, Bristol, United Kingdom), CGP 55,845 hydrochloride (benzyl-[(2 S)-3-[[(1 S)-1-(3,4-dichlorophenyl)ethyl]amino]-2-hydroxypropyl]phosphinic acid; hydrochloride; Hello Bio), Cyclopiazonic acid ((2R,3 S,9R)-5-acetyl-4-hydroxy-8,8-dimethyl-7,16-diazapentacyclo[9.6.1.02,9.03,7.015,18]octadeca-1(17),4,11(18),12,14-pentaen-6-one; Hello Bio), D-AP5 ((2R)-2-amino-5-phosphonopentanoic acid; Alomone Labs, Jerusalem, Israel), Norepinephrine bitartrate (4-[(1*R*)-2-amino-1-hydroxyethyl]benzene-1,2-diol;2,3-dihydroxybutanedioic acid; Merck KGaA, Darmstadt, Germany), Forskolin ([(3R,4aR,5 S,6 S,6aS,10 S,10aR,10bS)-3-ethenyl-6,10,10b-trihydroxy-3,4a,7,7,10a-pentamethyl-1-oxo-5,6,6a,8,9,10-hexahydro-2 H-benzo[f]chromen-5-yl] acetate; Cayman Chemical, Ann Arbor, Michigan, USA), Gabazine (4-[6-imino-3-(4-methoxyphenyl)pyridazin-1-yl]butanoic acid; Abcam), HC-067047 (2-methyl-1-(3-morpholin-4-ylpropyl)-5-phenyl-N-[3-(trifluoromethyl)phenyl]pyrrole-3-carboxamide; Calbiochem), ICI 118,551 hydrochloride ((2R,3 S)-1-[(7-methyl-2,3-dihydro-1 H-inden-4-yl)oxy]-3-(propan-2-ylamino)butan-2-ol; hydrochloride; Tocris Bioscience, Bristol, United Kingdom), Isoprenaline hydrochloride (4-[1-hydroxy-2-(propan-2-ylamino)ethyl]benzene-1,2-diol; hydrochloride; Abcam), NBQX (disodium;6-nitro-7-sulfamoylbenzo[f]quinoxaline-2,3-diolate; Alomone Labs), NKY80 (2-amino-7-(furan-2-yl)-7,8-dihydro-6 H-quinazolin-5-one; Tocris Bioscience), Prazosin hydrochloride ([4-(4-amino-6,7-dimethoxyquinazolin-2-yl)piperazin-1-yl]-(furan-2-yl)methanone; hydrochloride; Tocris Bioscience), Rauwolscine hydrochloride (methyl (1 S,15 S,18 S,19 S,20 S)-18-hydroxy-1,3,11,12,14,15,16,17,18,19,20,21-dodecahydroyohimban-19-carboxylate; hydrochloride; Tocris Bioscience), RS100329 hydochloride (5-methyl-3-[3-[4-[2-(2,2,2-trifluoroethoxy)phenyl]piperazin-1-yl]propyl]-1 H-pyrimidine-2,4-dione; hydrochloride; Tocris Bioscience), ST 034307 (6-chloro-2-(trichloromethyl)chromen-4-one; Tocris Bioscience), tetrodotoxin citrate ((1R,5R,6R,7R,9 S,11 S,12 S,13 S,14 S)-3-amino-14-(hydroxymethyl)-8,10-dioxa-2,4-diazatetracyclo[7.3.1.17,11.01,6]tetradec-3-ene-5,9,12,13,14-pentol; Hello Bio), Xylazine (N-(2,6-dimethylphenyl)-5,6-dihydro-4 H-1,3-thiazin-2-amine; hydrochloride; Merck). Stock solutions of drugs were prepared as stated by the manufacturer. Stocks solutions were diluted in ACSF and applied via the perfusion system.

### Preparation of acute brain slices

Mice were anesthetized with isoflurane (5% v/v in oxygen) and decapitated. OBs were quickly dissected in chilled Ca^2+^-reduced ACSF. 220 μm sagittal slices of OBs (Fig. 1B, C) were prepared using a vibratome (VT1200S, Leica, Bensheim, Germany), stored in ACSF at 30 °C for 30 min, and then kept at room temperature until the start of the experiment. Solutions were steadily gassed with carbogen (95% O_2_, 5% CO_2_).

### Confocal Imaging

After transferring slices to the recording chamber, imaging was performed with a confocal microscope (eC1, Nikon, Düsseldorf, Germany). Flamindo 2 was excited at 488 nm and emission detected from 500 to 530 nm. Images of astrocytes located in the glomerular and external plexiform layers (see Fig. 1C) [61] were acquired at a rate of one frame every 5 s. Noradrenergic agonists were applied for 30 s via the perfusion system, using a peristaltic pump and a pump speed of 2.35 ml/min. Antagonists were incubated for 10 min before agonists were applied, except inhibitors of adenylyl cyclases (ACs) and 2-APB which were incubated for 30 min. Similar settings were used to image intracellular Ca^2+^ using GCaMP6s. For simultaneous imaging of cAMP and Ca^2+^, Flamindo2 was excited at 488 nm and jRCaMP1b was excited at 543 nm and emission collected between 553 and 618 nm. For H^+^ (pH) measurements, acute slices were incubated with 10 µM pHrodo Red AM and 100 µM PowerLoad (both from Invitrogen, Carlsbad, CA, USA) for 30 min and the same optical parameters were used for pHrodo Red imaging as for jRCaMP1b.

### Immunohistochemistry

Immunohistological staining was performed as described before [62]. Brains were fixed in 4% formaldehyde in phosphate-buffered saline, pH 7.0 (stabilized with methanol, Roti-Histofix P087.4, Roth, Karlsruhe, Germany) for 1 h and washed three times in PBS (in mM): 130 NaCl, 7 Na_2_HPO_4_, 3 NaH_2_PO_4_, pH adjusted to 7.4. OBs were sliced into 150 μm sagittal slices by a vibratome (VT1000, Leica, Bensheim, Germany). Slices were incubated in blocking solution containing 5% normal goat serum (NGS, Cell Signaling Technology) and 0.5% Triton X-100 in PBS for 60 min at room temperature. Slices were incubated with primary antibodies for 48 h at room temperature diluted in primary antibody solution (blocking solution 1:10 in PBS), three times washed in PBS and subsequently incubated in secondary antibodies (dilution: 1:1000 in PBS) at 4 °C for 24 h. Afterwards slices were washed 3 X in PBS, and embedded on microscope slides with Shandon Immu-Mount (Thermo Fisher Scientific) and cover-slipped. Images were captured by confocal microscope (eC1, Nikon, Düsseldorf, Germany) and processed using Fiji ImageJ (http://imagej.org) and GIMP2.0 (https://www.gimp.org). The following antibodies were used: Rabbit anti-GFAP (1:500; Z0334 429-2; Dako, Agilent Technologies, Glostrup, Denmark), chicken anti-GFP (1:500; NB100-1614; Novus Biologicals, Centennial, Colorado, USA), rabbit anti-S100B (1:500; Z0311; Dako). Secondary antibodies: Goat anti-chicken Alexa 488 (1:1000; Invitrogen), goat anti-rabbit Alexa 555 (1:1000; Invitrogen). Nuclear stain: DAPI (5 µM; A1001 ; Applichem, Darmstadt, Germany).

### Data analysis and statistics

Imaging data was analyzed by marking cells as regions of interest (ROIs) in EZ-C1Viewer software (Nikon) and extracting data to Excel (Microsoft, USA) and Origin Pro 9.1 (OriginLab Corporation, Northampton, USA). The intensity of basal fluorescence F was set to 100% and relative changes of fluorescence were measured (ΔF). Signals with relative changes below 3% ΔF did not stand out significantly against noise and were excluded from further analysis. In contrast to GCaMP6s and pHrodo Red, which increase their fluorescence with an increasing concentration of the respective ion, an increase in the intracellular cAMP concentration is reflected by a decrease in Flamindo2 fluorescence [38] and we depict fluorescence traces inverted (-ΔF) to better illustrate actual cAMP changes. Traces represent one single exemplary cell. All values are stated as mean values ± standard error of the mean. The number n corresponds to the number of analyzed cells. Each set of experiments was carried out on brain slices prepared from at least three animals. A sigmoidal fit was used to analyze the dose-response relationship of NE-evoked cAMP transients (Origin Pro 9.1). The following tests were used for evaluation of statistical significance: Outliers were identified and dismissed by Grubbs test. Wilcoxon signed rank test was used for paired data, Mann-Whitney-U test for independent data. For comparisons involving more than two groups, the Kruskal-Wallis ANOVA followed by Dunn´s post hoc test was applied for independent data, and the Friedmann ANOVA followed by Wilcoxon signed-rank post hoc test for paired data. Significant differences were assumed with error probabilities *p* < 0.05 (*p < 0.05, **p < 0.01, ***p < 0.001).

## Supplementary Information

Supplementary Material 1.

## Data Availability

The data that support the findings of this study are available from the corresponding author, CL, upon reasonable request.

## References

[1] Fuller RW. Pharmacology of brain epinephrine neurons. Annu Rev Pharmacol Toxicol. 1982;22:31–55. 6805416 10.1146/annurev.pa.22.040182.000335

[2] Shipley MT, Halloran FJ, de la Torre J. Surprisingly rich projection from locus coeruleus to the olfactory bulb in the rat. Brain Res. 1985;329:294–9. 3978450 10.1016/0006-8993(85)90537-2

[3] Linster C, Midroit M, Forest J, Thenaisie Y, Cho C, Richard M, Didier A, Mandairon N. Noradrenergic Activity in the Olfactory Bulb Is a Key Element for the Stability of Olfactory Memory. J Neurosci. 2020;40:9260–71. 33097638 10.1523/JNEUROSCI.1769-20.2020 PMC7687066

[4] Linster C, Nai Q, Ennis M. Nonlinear effects of noradrenergic modulation of olfactory bulb function in adult rodents. J Neurophysiol. 2011;105:1432–43. 21273323 10.1152/jn.00960.2010 PMC3075300

[5] Paredes-Rodriguez E, Vegas-Suarez S, Morera-Herreras T, De Deurwaerdere P, Miguelez C. The Noradrenergic System in Parkinson’s Disease. Front Pharmacol. 2020;11:435. 32322208 10.3389/fphar.2020.00435 PMC7157437

[6] Zhang YM, Qi YB, Gao YN, Chen WG, Zhou T, Zang Y, Li J. Astrocyte metabolism and signaling pathways in the CNS. Front Neurosci. 2023;17:1217451. 37732313 10.3389/fnins.2023.1217451 PMC10507181

[7] Escartin C, Galea E, Lakatos A, O’Callaghan JP, Petzold GC, Serrano-Pozo A, Steinhauser C, Volterra A, Carmignoto G, Agarwal A, et al. Reactive astrocyte nomenclature, definitions, and future directions. Nat Neurosci. 2021;24:312–25. 33589835 10.1038/s41593-020-00783-4 PMC8007081

[8] Lohr C. Role of P2Y receptors in astrocyte physiology and pathophysiology. Neuropharmacology. 2023;223:109311. 36328064 10.1016/j.neuropharm.2022.109311

[9] Verkhratsky A, Nedergaard M. Physiology of Astroglia. Physiol Rev. 2018;98:239–389. 29351512 10.1152/physrev.00042.2016 PMC6050349

[10] Ahlquist RP. A study of the adrenotropic receptors. Am J Physiol. 1948;153:586–600. 18882199 10.1152/ajplegacy.1948.153.3.586

[11] Lands AM, Arnold A, McAuliff JP, Luduena FP, Brown TG Jr. Differentiation of receptor systems activated by sympathomimetic amines. Nature. 1967;214:597–8. 6036174 10.1038/214597a0

[12] Strosberg AD. Structure, function, and regulation of adrenergic receptors. Protein Sci. 1993;2:1198–209. 8401205 10.1002/pro.5560020802 PMC2142449

[13] Neves SR, Ram PT, Iyengar R. G protein pathways. Science. 2002;296:1636–9. 12040175 10.1126/science.1071550

[14] Wahis J, Holt MG. Astrocytes, Noradrenaline, α1-Adrenoreceptors, and Neuromodulation: Evidence and Unanswered Questions. Front Cell Neurosci. 2021;15:645691. 33716677 10.3389/fncel.2021.645691 PMC7947346

[15] Fischer T, Prey J, Eschholz L, Rotermund N, Lohr C. Norepinephrine-Induced Calcium Signaling and Store-Operated Calcium Entry in Olfactory Bulb Astrocytes. Front Cell Neurosci. 2021;15:639754. 33833669 10.3389/fncel.2021.639754 PMC8021869

[16] Chen J, Ding Q, An L, Wang H. Ca(2+)-stimulated adenylyl cyclases as therapeutic targets for psychiatric and neurodevelopmental disorders. Front Pharmacol. 2022;13:949384. 36188604 10.3389/fphar.2022.949384 PMC9523369

[17] Horvat A, Zorec R, Vardjan N. Adrenergic stimulation of single rat astrocytes results in distinct temporal changes in intracellular Ca(2+) and cAMP-dependent PKA responses. Cell Calcium. 2016;59:156–63. 26794933 10.1016/j.ceca.2016.01.002

[18] Oe Y, Wang X, Patriarchi T, Konno A, Ozawa K, Yahagi K, Hirai H, Tsuboi T, Kitaguchi T, Tian L, et al. Distinct temporal integration of noradrenaline signaling by astrocytic second messengers during vigilance. Nat Commun. 2020;11:471. 31980655 10.1038/s41467-020-14378-x PMC6981284

[19] von Kalben L, Sauer J, Gee C, Hirnet D, Lohr C. Dopaminergic cAMP signaling in mouse olfactory bulb astrocytes. Neurochem Int. 2024;179:105828. 39134121 10.1016/j.neuint.2024.105828

[20] Theparambil SM, Kopach O, Braga A, Nizari S, Hosford PS, Sagi-Kiss V, Hadjihambi A, Konstantinou C, Esteras N, Del Gutierrez A, et al. Adenosine signalling to astrocytes coordinates brain metabolism and function. Nature. 2024;632:139–46. 38961289 10.1038/s41586-024-07611-w PMC11291286

[21] Wendlandt M, Kürten AJ, Beiersdorfer A, Schubert C, Samad-Yazdtchi K, Sauer J, Pinto MC, Schulz K, Friese MA, Gee CE, et al. A(2A) adenosine receptor-driven cAMP signaling in olfactory bulb astrocytes is unaffected in experimental autoimmune encephalomyelitis. Front Immunol. 2023;14:1273837. 38077336 10.3389/fimmu.2023.1273837 PMC10701430

[22] Vardjan N, Chowdhury HH, Horvat A, Velebit J, Malnar M, Muhič M, Kreft M, Krivec ŠG, Bobnar ST, Miš K, et al. Enhancement of Astroglial Aerobic Glycolysis by Extracellular Lactate-Mediated Increase in cAMP. Front Mol Neurosci. 2018;11:148. 29867342 10.3389/fnmol.2018.00148 PMC5953330

[23] Choi HB, Gordon GR, Zhou N, Tai C, Rungta RL, Martinez J, Milner TA, Ryu JK, McLarnon JG, Tresguerres M, et al. Metabolic communication between astrocytes and neurons via bicarbonate-responsive soluble adenylyl cyclase. Neuron. 2012;75:1094–104. 22998876 10.1016/j.neuron.2012.08.032 PMC3630998

[24] Hasel P, Dando O, Jiwaji Z, Baxter P, Todd AC, Heron S, Márkus NM, McQueen J, Hampton DW, Torvell M, et al. Neurons and neuronal activity control gene expression in astrocytes to regulate their development and metabolism. Nat Commun. 2017;8:15132. 28462931 10.1038/ncomms15132 PMC5418577

[25] Vittani M, Herlo R, Wang X, Christensen MDB, Vo CT, Mishima T, Kusk P, Konno A, Hirai H, Tsuboi T, et al. Cerebral blood flow is modulated by astrocytic cAMP elevation independently of IP(3)R2-mediated Ca(2+) signaling in mice. Proc Natl Acad Sci U S A. 2025;122:e2422069122. 40591593 10.1073/pnas.2422069122 PMC12260423

[26] Vardjan N, Zorec R. Excitable Astrocytes: Ca(2+)- and cAMP-Regulated Exocytosis. Neurochem Res. 2015;40:2414–24. 25732760 10.1007/s11064-015-1545-x

[27] Sitjà-Roqueta L, Ngum NM, Zherebtsov EA, Küçükerden M, Givehchi M, Bova V, Delicata F, Anaya-Cubero E, Santamaria E, Fernández-Irigoyen J, et al. Photoactivated adenylyl cyclase in cortical astrocytes promotes synaptic potentiation and reveals alterations in Huntington’s disease. iScience. 2025;28:113640. 41146711 10.1016/j.isci.2025.113640 PMC12554215

[28] Shigetomi E, Koizumi S. The role of astrocytes in behaviors related to emotion and motivation. Neurosci Res. 2023;187:21–39. 36181908 10.1016/j.neures.2022.09.015

[29] Zhou Z, Okamoto K, Onodera J, Hiragi T, Andoh M, Ikawa M, Tanaka KF, Ikegaya Y, Koyama R. Astrocytic cAMP modulates memory via synaptic plasticity. Proc Natl Acad Sci U S A. 2021;118.e2016584118. 10.1073/pnas.2016584118 PMC7826339 33452135

[30] Zorec R, Horvat A, Vardjan N, Verkhratsky A. Memory Formation Shaped by Astroglia. Front Integr Neurosci. 2015;9:56. 26635551 10.3389/fnint.2015.00056 PMC4648070

[31] Devasani K, Yao Y. Expression and functions of adenylyl cyclases in the CNS. Fluids Barriers CNS. 2022;19:23. 35307032 10.1186/s12987-022-00322-2 PMC8935726

[32] Sadana R, Dessauer CW. Physiological roles for G protein-regulated adenylyl cyclase isoforms: insights from knockout and overexpression studies. Neurosignals. 2009;17:5–22. 18948702 10.1159/000166277 PMC2790773

[33] Sobolczyk M, Boczek T. Astrocytic Calcium and cAMP in Neurodegenerative Diseases. Front Cell Neurosci. 2022;16:889939. 35663426 10.3389/fncel.2022.889939 PMC9161693

[34] Li HL, Verhoeven A, Elferink RO. The role of soluble adenylyl cyclase in sensing and regulating intracellular pH. Pflugers Arch. 2024;476:457–65. 38581526 10.1007/s00424-024-02952-x PMC11006738

[35] Rotermund N, Schulz K, Hirnet D, Lohr C. Purinergic Signaling in the Vertebrate Olfactory System. Front Cell Neurosci. 2019;13:112. 31057369 10.3389/fncel.2019.00112 PMC6477478

[36] Gao V, Suzuki A, Magistretti PJ, Lengacher S, Pollonini G, Steinman MQ, Alberini CM. Astrocytic β2-adrenergic receptors mediate hippocampal long-term memory consolidation. Proc Natl Acad Sci U S A. 2016;113:8526–31. 27402767 10.1073/pnas.1605063113 PMC4968707

[37] Nomura S, Bouhadana M, Morel C, Faure P, Cauli B, Lambolez B, Hepp R. Noradrenalin and dopamine receptors both control cAMP-PKA signaling throughout the cerebral cortex. Front Cell Neurosci. 2014;8:247. 25191229 10.3389/fncel.2014.00247 PMC4140213

[38] Odaka H, Arai S, Inoue T, Kitaguchi T. Genetically-encoded yellow fluorescent cAMP indicator with an expanded dynamic range for dual-color imaging. PLoS ONE. 2014;9:e100252. 24959857 10.1371/journal.pone.0100252 PMC4069001

[39] Lee Y, Messing A, Su M, Brenner M. GFAP promoter elements required for region-specific and astrocyte-specific expression. Glia. 2008;56:481–93. 18240313 10.1002/glia.20622

[40] Singaravelu K, Lohr C, Deitmer JW. Regulation of store-operated calcium entry by calcium-independent phospholipase A2 in rat cerebellar astrocytes. J Neurosci. 2006;26:9579–92. 16971542 10.1523/JNEUROSCI.2604-06.2006 PMC6674595

[41] Stavermann M, Buddrus K, St John JA, Ekberg JA, Nilius B, Deitmer JW, Lohr C. Temperature-dependent calcium-induced calcium release via InsP3 receptors in mouse olfactory ensheathing glial cells. Cell Calcium. 2012;52:113–23. 22633850 10.1016/j.ceca.2012.04.017

[42] Doengi M, Hirnet D, Coulon P, Pape HC, Deitmer JW, Lohr C. GABA uptake-dependent Ca(2+) signaling in developing olfactory bulb astrocytes. Proc Natl Acad Sci U S A. 2009;106:17570–5. 19805126 10.1073/pnas.0809513106 PMC2765163

[43] Bylund DB, Eikenberg DC, Hieble JP, Langer SZ, Lefkowitz RJ, Minneman KP, Molinoff PB, Ruffolo RR Jr., Trendelenburg U. International Union of Pharmacology nomenclature of adrenoceptors. Pharmacol Rev. 1994;46:121–36. 7938162

[44] Hieble JP, Bylund DB, Clarke DE, Eikenburg DC, Langer SZ, Lefkowitz RJ, Minneman KP, Ruffolo RR Jr. International Union of Pharmacology. X. Recommendation for nomenclature of alpha 1-adrenoceptors: consensus update. Pharmacol Rev. 1995;47:267–70. 7568329

[45] Brand CS, Hocker HJ, Gorfe AA, Cavasotto CN, Dessauer CW. Isoform selectivity of adenylyl cyclase inhibitors: characterization of known and novel compounds. J Pharmacol Exp Ther. 2013;347:265–75. 24006339 10.1124/jpet.113.208157 PMC3807061

[46] Brust TF, Alongkronrusmee D, Soto-Velasquez M, Baldwin TA, Ye Z, Dai M, Dessauer CW, van Rijn RM, Watts VJ. Identification of a selective small-molecule inhibitor of type 1 adenylyl cyclase activity with analgesic properties. Sci Signal. 2017;10.eaah5381. 10.1126/scisignal.aah5381 PMC5734633 28223412

[47] Zimnik NC, Treadway T, Smith RS, Araneda RC. α(1A)-Adrenergic regulation of inhibition in the olfactory bulb. J Physiol. 2013;591:1631–43. 23266935 10.1113/jphysiol.2012.248591 PMC3624843

[48] Baker JG, Summers RJ. Adrenoceptors: Receptors, Ligands and Their Clinical Uses, Molecular Pharmacology and Assays. Handb Exp Pharmacol. 2024;285:55–145. 38926158 10.1007/164_2024_713

[49] Mather M, Clewett D, Sakaki M, Harley CW. Norepinephrine ignites local hotspots of neuronal excitation: How arousal amplifies selectivity in perception and memory. Behav Brain Sci. 2016;39:e200. 26126507 10.1017/S0140525X15000667 PMC5830137

[50] Ramos BP, Arnsten AF. Adrenergic pharmacology and cognition: focus on the prefrontal cortex. Pharmacol Ther. 2007;113:523–36. 17303246 10.1016/j.pharmthera.2006.11.006 PMC2151919

[51] Rosenberg MF, Godoy MI, Wade SD, Paredes MF, Zhang Y, Molofsky AV. β-Adrenergic Signaling Promotes Morphological Maturation of Astrocytes in Female Mice. J Neurosci. 2023;43:8621–36. 37845031 10.1523/JNEUROSCI.0357-23.2023 PMC10727121

[52] Chen X, Meroueh M, Mazur G, Rouse E, Hundal KS, Stamatkin CW, Obukhov AG. Phenylephrine, a common cold remedy active ingredient, suppresses uterine contractions through cAMP signalling. Sci Rep. 2018;8:11666. 30076382 10.1038/s41598-018-30094-5 PMC6076280

[53] Onda T, Hashimoto Y, Nagai M, Kuramochi H, Saito S, Yamazaki H, Toya Y, Sakai I, Homcy CJ, Nishikawa K, Ishikawa Y. Type-specific regulation of adenylyl cyclase. Selective pharmacological stimulation and inhibition of adenylyl cyclase isoforms. J Biol Chem. 2001;276:47785–93. 11602596 10.1074/jbc.M107233200

[54] Jaiswal BS, Conti M. Calcium regulation of the soluble adenylyl cyclase expressed in mammalian spermatozoa. Proc Natl Acad Sci U S A. 2003;100:10676–81. 12958208 10.1073/pnas.1831008100 PMC196863

[55] Muglia LM, Schaefer ML, Vogt SK, Gurtner G, Imamura A, Muglia LJ. The 5’-flanking region of the mouse adenylyl cyclase type VIII gene imparts tissue-specific expression in transgenic mice. J Neurosci. 1999;19:2051–8. 10066258 10.1523/JNEUROSCI.19-06-02051.1999 PMC6782540

[56] Sardar D, Cheng YT, Woo J, Choi DJ, Lee ZF, Kwon W, Chen HC, Lozzi B, Cervantes A, Rajendran K, et al. Induction of astrocytic Slc22a3 regulates sensory processing through histone serotonylation. Science. 2023;380:eade0027. 37319217 10.1126/science.ade0027 PMC10874521

[57] Jang MJ, Coughlin GM, Jackson CR, Chen X, Chuapoco MR, Vendemiatti JL, Wang AZ, Gradinaru V. Spatial transcriptomics for profiling the tropism of viral vectors in tissues. Nat Biotechnol. 2023;41:1272–86. 36702899 10.1038/s41587-022-01648-w PMC10443732

[58] Chan KY, Jang MJ, Yoo BB, Greenbaum A, Ravi N, Wu WL, Sánchez-Guardado L, Lois C, Mazmanian SK, Deverman BE, Gradinaru V. Engineered AAVs for efficient noninvasive gene delivery to the central and peripheral nervous systems. Nat Neurosci. 2017;20:1172–9. 28671695 10.1038/nn.4593 PMC5529245

[59] Chen TW, Wardill TJ, Sun Y, Pulver SR, Renninger SL, Baohan A, Schreiter ER, Kerr RA, Orger MB, Jayaraman V, et al. Ultrasensitive fluorescent proteins for imaging neuronal activity. Nature. 2013;499:295–300. 23868258 10.1038/nature12354 PMC3777791

[60] Mori T, Tanaka K, Buffo A, Wurst W, Kühn R, Götz M. Inducible gene deletion in astroglia and radial glia–a valuable tool for functional and lineage analysis. Glia. 2006;54:21–34. 16652340 10.1002/glia.20350

[61] Lohr C, Grosche A, Reichenbach A, Hirnet D. Purinergic neuron-glia interactions in sensory systems. Pflugers Arch. 2014;466:1859–72. 24705940 10.1007/s00424-014-1510-6

[62] Beiersdorfer A, Wolburg H, Grawe J, Scheller A, Kirchhoff F, Lohr C. Sublamina-specific organization of the blood brain barrier in the mouse olfactory nerve layer. Glia. 2020;68:631–45. 31696993 10.1002/glia.23744

