# Supplementary material for: Crosstalk of noradrenergic Ca2+ and cAMP signaling in astrocytes of the murine olfactory bulb
Source: Cell Commun Signal. 2026 Jul 11;24:397. doi: 10.1186/s12964-026-03055-z (PMC13355343; doi:10.1186/s12964-026-03055-z)
Supplement: Supplementary file 1 — Supplementary Material 1. [file 12964_2026_3055_MOESM1_ESM.pdf]

Supplementary Materials for

Crosstalk of noradrenergic Ca^{2+} and cAMP signaling in astrocytes of the murine olfactory bulb

Jessica Sauer, Antonia Beiersdorfer, Franz Lennart Schmidt, Mathias Nordbeck, Oana Constantin, Daniela Hirnet, Christine Gee, Christian Lohr*

*Corresponding author: christian.lohr@uni-hamburg.de

Figure s1
Figure s2
Figure s3
Figure s4
Figure s5
Figure s6
Figure s7
Figure s8
Figure s9
Table s1
Table s2

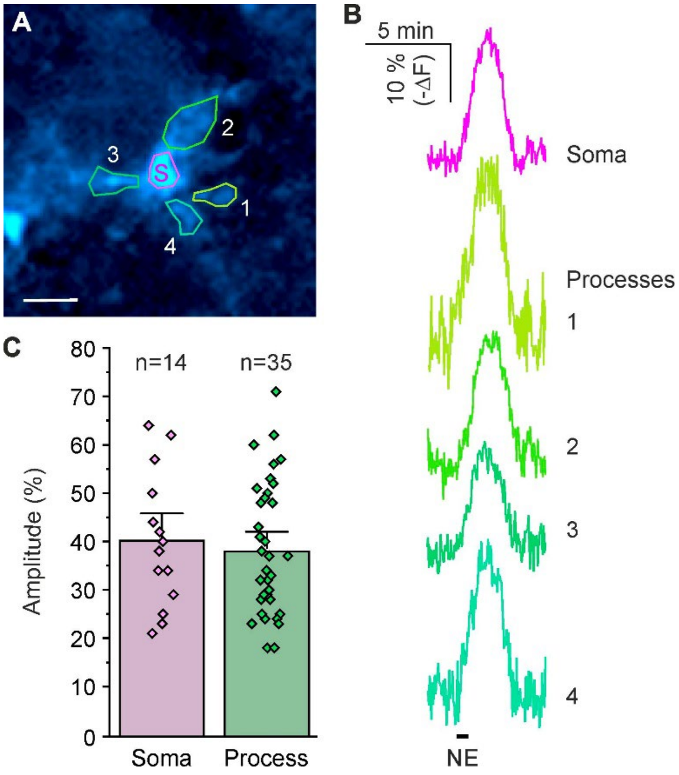

Fig. s1. NE-evoked changes in cAMP in soma and processes of an astrocyte. (**A**) ROIs to analyze fluorescence changes in the soma (S, magenta) and processes (1-4, green) of an individual olfactory bulb astrocyte expressing Flamindo2. (**B**) Flamindo2 fluorescence traces of cAMP signals as recorded in the soma and processes of the astrocyte depicted in (**A**). (**C**) Analysis of the relative fluorescence changes revealed no statistical difference ($p = 0.64$, Mann-Whitney U-Test) between soma (40.2 ± 3.7 % -ΔF) and processes (38.5 ± 2.3 % -ΔF). Data from 14 cell/6 mice.

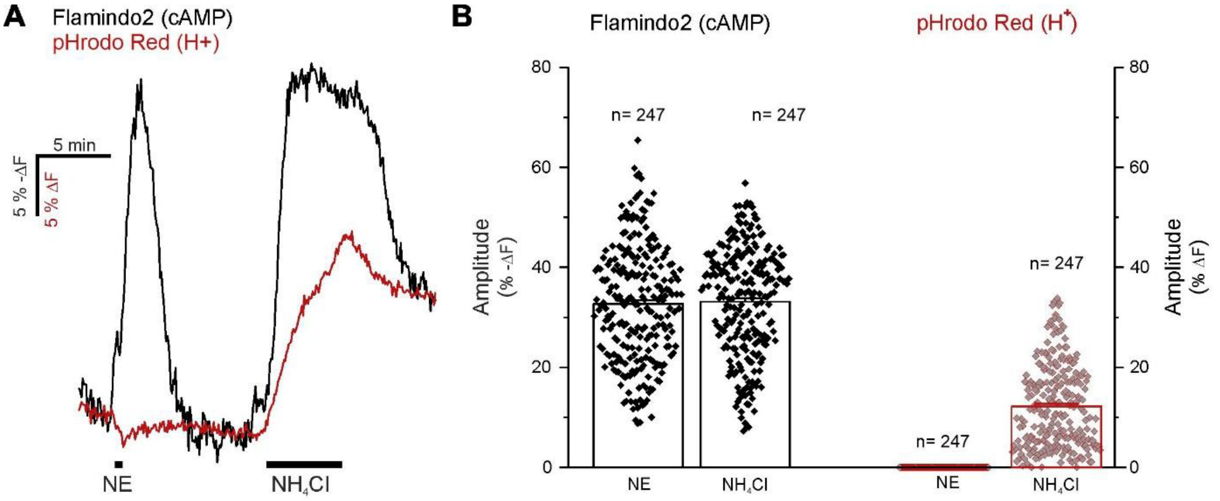

Fig. s2. Effect of pH changes on Flamindo2 fluorescence. (**A**) Changes in cAMP (black) and H^+ (red) concentrations induced by norepinephrine (NE) and NH_4Cl. Note that the Flamindo2 trace is inverted (-ΔF) and Flamindo2 fluorescence decreases upon acidification by NH_4Cl. (**B**) Quantification of cAMP and H^+ changes. The results demonstrate pH sensitivity of Flamindo2, however, application of NE fails to evoke pH shifts, indicating that NE-evoked changes in Flamindo2 fluorescence are not pH-dependent. Data from 4 mice.

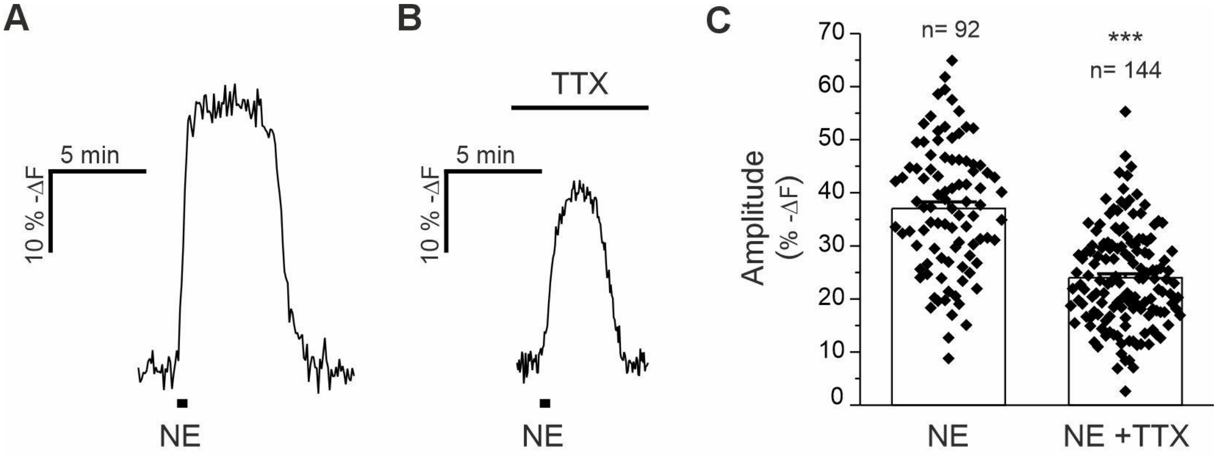

Fig. s3. Tetrodotoxin (TTX) reduces NE-evoked cAMP responses in OB astrocytes. (A) Bath application of norepinephrine (NE, 10 µM) for 30 s resulted in transient increases in cAMP in the absence (control) and in (B) the presence of 0.5 µM TTX. (C) NE-evoked increases in cAMP were significantly reduced by TTX. ***$p < 0.001$, Mann-Whitney U-test; data from 6 mice for control (NE), 6 mice for TTX (NE + TTX).

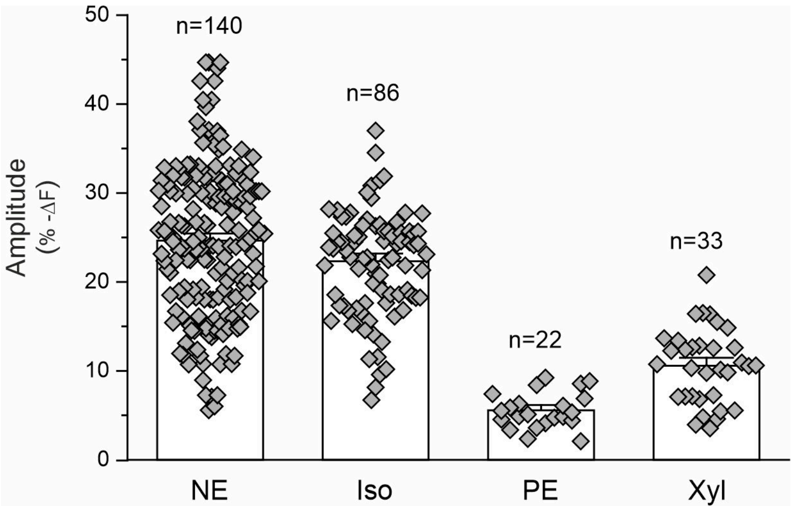

Fig. s4. Amplitudes of cAMP responses evoked by various adrenergic agonists in OB astrocytes. Bath application of norepinephrine (NE, 10 µM; data from 6 mice), isoprenaline (Iso, 100 µM; data from 3 mice), phenylephrine (PE, 100 µM; data from 4 mice) and xylazine (Xyl, 80 µM; data from 3 mice) evoked increases in the cAMP concentration.

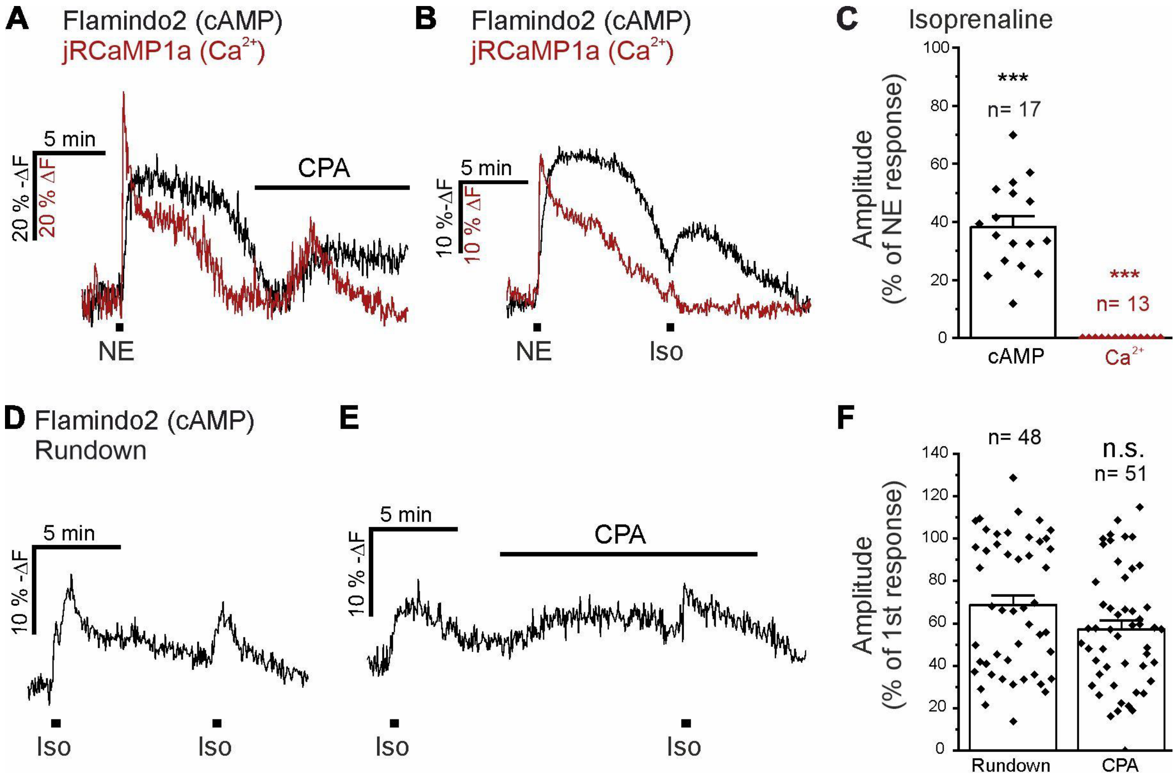

Fig. s5. Cyclopiazonic acid and isoprenaline induced cAMP signals in olfactory bulb astrocytes. (**A**) Depletion of intracellular Ca^{2+} stores by cyclopiazonic acid is visible by a moderate increase in jRCaMP1a fluorescence (red trace) and accompanied by an increase in Flamindo2 fluorescence (black trace), indicating a rise in cAMP. Norepinephrine (NE, 10 µM) was applied as a control to test for vital Ca^{2+} and cAMP signaling. (**B**) NE evoked both cAMP (black trace) and Ca^{2+} signals (red trace), whereas isoprenaline (Iso, 100 µM) induced only cAMP signals. (**C**) Analysis of the Iso-induced cAMP and Ca^{2+} signals, normalized to the amplitude of the NE-evoked response which was set to 100 %. ***$p < 0.001$, Mann-Whitney-U test. Rundown control: Data from 3 mice Flamindo2, 3 mice Flamindo2 + jRCaMP1a; Iso: data from 2 mice Flamindo2 + jRCaMP1a. (**D**) cAMP signals evoked by repetitive application of Iso as a rundown experiment. (**E**) Effect of 20 µM cyclopiazonic acid (CPA) on Iso-evoked cAMP signals. (**F**) In the presence of CPA, Iso-evoked cAMP responses were not significantly altered compared to the corresponding rundown experiment. n.s. not significant, Mann-Whitney-U test; rundown: data from 3 mice; CPA: data from 3 mice.

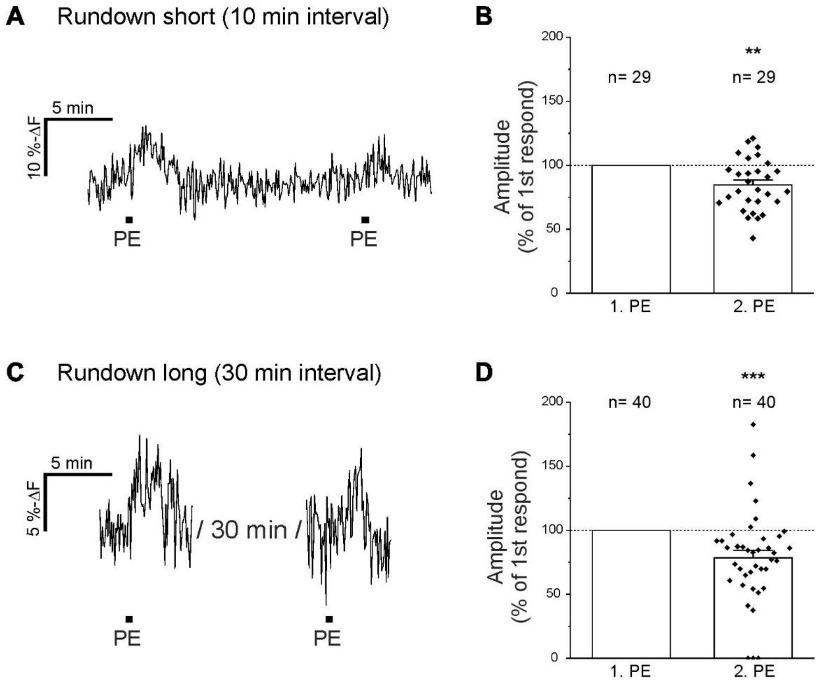

Fig. s6 Phenylephrine rundown experiments. (**A**) Phenylephrine (PE)-induced cAMP signals evoked by two PE applications with intervals of 10 minutes (rundown short). **(B)** A second application of PE evoked significantly smaller responses compared to the first application. **p < 0.01, Mann-Whitney-U test, data from 3 mice. (**C**) PE applications with intervals of 30 minutes (rundown long). **(B)** A second application of PE evoked significantly smaller responses compared to the first application. ***p < 0.001, Mann-Whitney-U test; data from 7 mice.

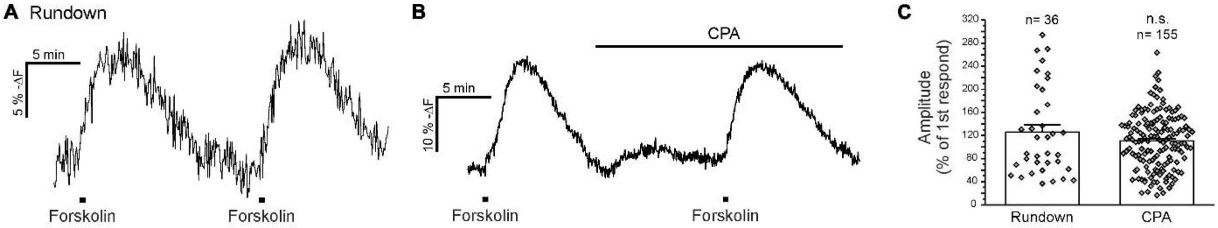

Fig. s7 Cyclopiazonic acid has no effect on adenylyl cyclase activity. (**A**) Astrocytic cAMP signals evoked by repetitive application of 3 µM forskolin. (**B**) Forskolin-evoked cAMP transients before and in the presence of 20 µM cyclopiazonic acid (CPA). (**C**) CPA has no significant effect on forskolin-evoked cAMP increases n.s. not significant, Mann-Whitney-U test, data from 4 mice for rundown, 5 mice for CPA.

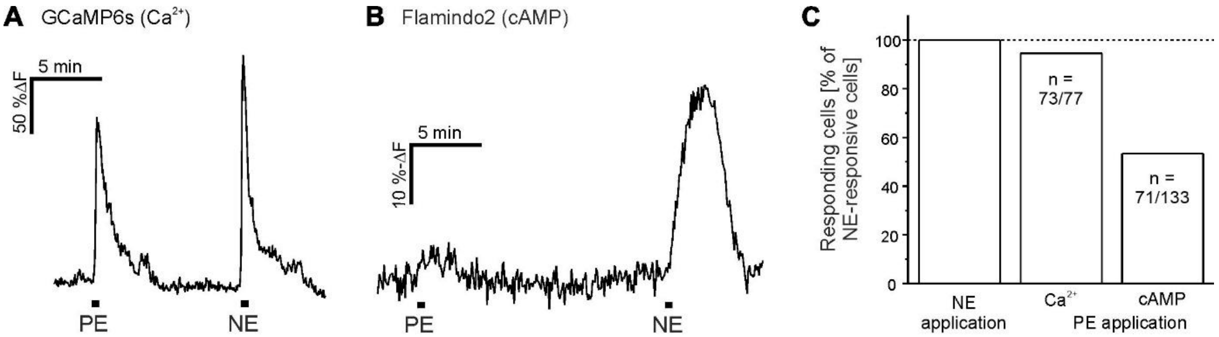

Fig. s8 Ca^{2+} and cAMP signaling induced by phenylephrine and norepinephrine. (**A**) Phenylephrine (PE)- and norepinephrine (NE)-induced Ca^{2+} and (**B**) cAMP signals. (**C**) Fraction of responding astrocytes. While virtually all astrocytes responded to NE and PE with Ca^{2+} transients, only 53 % of astrocytes (71 out of 133) responded to PE application with an increase in cAMP. Data from 3 mice for Ca^{2+}, 3 mice for cAMP.

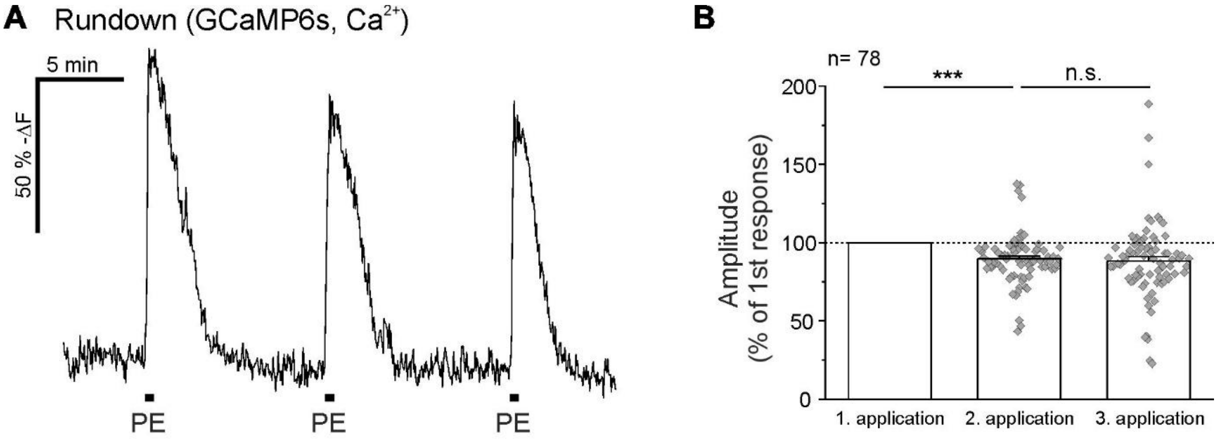

Fig. s9 Rundown of three phenylephrine (PE)-induced Ca^{2+} signals. (A) Ca^{2+} signals in olfactory bulb astrocytes evoked by repetitive PE-applications. **(B)** Analysis of the Ca^{2+} signal amplitudes, normalized to the amplitude of the first response. n.s.=not significant, ***, $p < 0.001$. Friedmann ANOVA and Wilcoxon signed-rank post hoc test, data from 4 mice.

Table s1. Amplitudes of norepinephrine-induced cAMP signals in astrocytes at concentrations ranging from 1–30 µM (raw values for dose-response curve in Fig.1J). Data from 3 mice.

**Concentration norepinephrine**	**Mean amplitudes ± SEM** (% -ΔF)	**n**
1 µM	6.1 ± 0.8	88
3 µM	23.7 ± 1.1	88
10 µM	27.4 ± 0.9	88
30 µM	31.0 ± 0.9	88

Table s2. Amplitudes of phenylephrine-induced cAMP signals in astrocytes at concentrations ranging from 1–300 µM (raw values for dose-response curve in Fig. 4B). Data from 6 mice.

**Concentration phenylephrine**	**Mean amplitudes ± SEM** (% -ΔF)	**n**
1 µM	3.7 ± 0.5	61
3 µM	6.3 ± 0.3	84
10 µM	6.6 ± 0.2	112
30 µM	7.7 ± 0.4	80
100 µM	8.3 ± 0.5	57
300 µM	8.7 ± 0.7	29
